# Lost in Translation: Simulation-Informed Bayesian Inference Improves Characterization of Molecular Motion from Neutron Scattering

**DOI:** 10.1021/acs.jpclett.6c01399

**Published:** 2026-07-09

**Authors:** Harry Richardson, Kit McColl, Gøran J. Nilsen, Jeff Armstrong, Andrew R. McCluskey

**Affiliations:** † Centre for Computational Chemistry, School of Chemistry, 1980 University of Bristol, Cantock’s Close, Bristol BS8 1TS, U.K.; ‡ Department of Materials, University of Oxford, Parks Road, Oxford OX1 3PH, U.K.; § 601217 The Faraday Institution, Quad One, Harwell Science and Innovation Campus, Didcot OX11 0RA, U.K.; ∥ 120797 ISIS Pulsed Neutron and Muon Facility, Rutherford Appleton Laboratory, Didcot OX11 0QX, U.K.; ⊥ Department of Mathematics and Physics, University of Stavanger, 4036 Stavanger, Norway; # Department of Chemistry, University of Bath, Claverton Down, Bath BA2 7AY, U.K.; 7 Diamond Light Source, Harwell Campus, Didcot OX11 0DE, U.K.

## Abstract

Quasi-elastic neutron scattering (QENS) probes atomic and molecular motion on length and time scales central to catalysis, energy materials, and gas adsorption. However, conventional analytical fitting of QENS spectra often fails to uniquely determine the underlying dynamics. The flexibility of simplified line-shape models can make spectra generated by distinct physical processes statistically indistinguishable, leading to an ambiguous or inaccurate mechanistic interpretation. By integrating molecular dynamics simulations, physically derived *Q*-dependent scattering models, Bayesian model discrimination, and polarization analysis, we demonstrate that QENS can, for the first time, resolve anisotropic rotational motion in liquid benzene, a prototypical aromatic molecule relevant to microporous catalysis. The extracted spinning and tumbling diffusion coefficients suggest stronger anisotropy than previously recognized. This integrated, Bayesian evidence-based analytical framework defines a new paradigm for QENS, enabling direct resolution of the rotational and translational dynamics that govern molecular interactions and transport: the fundamental processes and rate-limiting steps in confined hydrocarbon catalysis.

## Graphical abstract

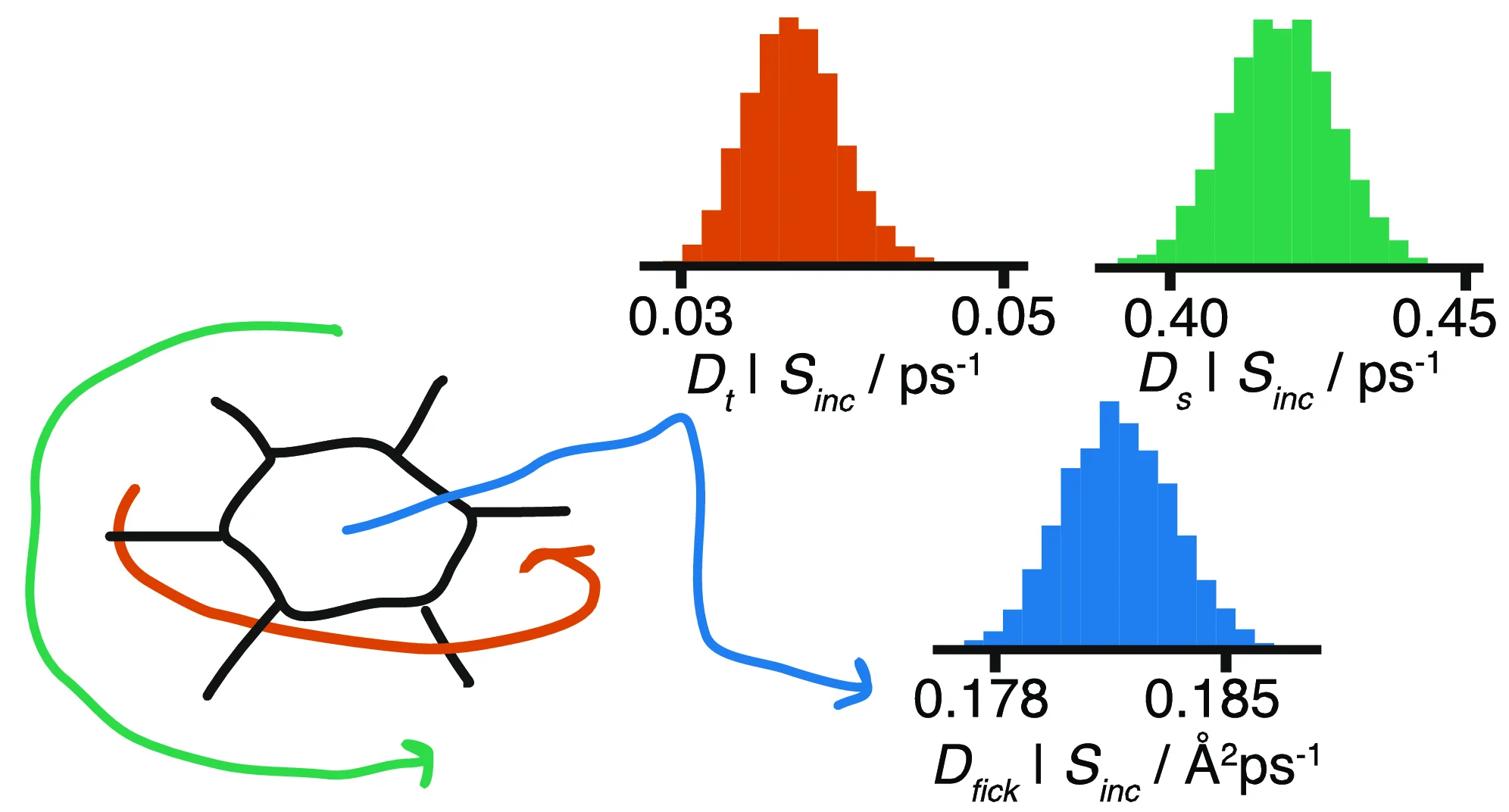

Progress in catalysis and materials chemistry relies on resolving molecular motion under confinement and in complex environments. Quasi-elastic neutron scattering (QENS), owing to its exceptional sensitivity to hydrogen, directly probes these processes over angstrom to nanometer length scales and subpicosecond to nanosecond time scales, precisely matching the spatial and temporal regimes that govern bonding interactions and molecular transport in catalytic and functional materials.^1–3^ Confined translational and rotational diffusion is often the key bottleneck in the catalytic efficiency of these materials. Accurate and atomistically resolved descriptions of these molecular motions, therefore, drive the design principles for higher-performing catalysts.^4^ For example, high rotation rates in catalytic pores have been associated with reduced catalytic efficiency, as the reactants cannot translate easily through narrow openings.^5^ More generally, QENS is widely applied across the physical sciences, where it can probe ionic migration,^6^ elucidate the diffusion of water through microporous polymer membranes,^7^ and study protein dynamics in solution.^8^

In principle, QENS enables the quantification of molecular rotation and translation by measuring the total dynamic structure factor, *S*(*Q*, ω), as a function of wavevector, *Q*, and energy transfer, ω, which fully encodes the spatial and temporal atomic correlations of the system. The total dynamic structure factor is the sum of coherent and incoherent components; the incoherent part, *S*_inc_(*Q*, ω), describes only the self-dynamics of atoms, while the coherent signal also contains information about collective dynamics.^8,9^ As is the case with other scattering methods, the inversion of the dynamic structure factor from the unintuitive momentum and frequency spaces to a more relatable spatial and temporal description is often not straightforward.^10^ The system’s dynamics are encoded in the line shape of the ω-dependent QENS spectra, which is convoluted with the instrument-specific spectral resolution. The evolution of the line shape with *Q* then describes the contributions from the system’s rotational and translational processes.

Typically, QENS spectra are analyzed by fitting a selected number of Lorentzian functions independently at each *Q*. The extracted line widths and amplitudes are then interpreted in terms of their *Q* dependence to infer the underlying rotational and translational mechanisms. This approach yields a high-dimensional, poorly constrained parameter space that can easily lead to “statistical bottlenecks” and degenerate descriptions of the same data,^11^ which can be fitted by multiple conflicting models, thereby undermining the physical reliability of the results. Furthermore, QENS analysis often relies on oversimplified models that potentially distort the dynamical behavior of the experimental system,^12^ making it challenging and subjective to draw physically meaningful interpretations. Finally, in the analysis of hydrogenous systems, it is typically presumed that the total dynamic structure factor consists of purely incoherent scattering, due to the large incoherent scattering cross section of ^1^H. However, the development of QENS with polarization analysis (p-QENS) has shown that coherent contributions can remain significant, and neglecting them can compromise the inferred dynamics.^8^

As catalytic and functional materials and processes become more complex, rigorous discrimination between competing transport mechanisms becomes essential. Addressing model degeneracy, global *Q* constraints, and coherent scattering effects within a unified framework is therefore necessary to obtain physically reliable insight from QENS measurements. Previous work has attempted to address these issues individually,^3,13–15^ but no prior approach has addressed them simultaneously. In this work, we overcome these challenges by showing how it is possible (i) to develop and use accurate analysis models, based on the physics and chemistry of our system, (ii) to reduce the number of fitting parameters by constraining the fit as a function of *Q*, (iii) to empirically compare different *Q*-dependent models, using Bayesian inference, and (iv) to address the presence of coherent scattering by taking advantage of p-QENS. To implement this framework, we use insights from molecular dynamics (MD) simulations to guide the interpretation of QENS spectra of liquid benzene. Benzene is used as a test case because, despite its structural simplicity, its complex dynamics, including multiple rotational modes,^16,17^ remain poorly characterized. Our approach is demonstrated through a state-of-the-art study in which the complex dynamics, and the rotational modes of liquid benzene, are quantified. Predictions from MD simulations are used to inform experimental design and guide QENS analysis. Finally, we show that this comprehensive modeling–theory combination can extract previously unobtained information about the dynamics in this classic, well-studied system.^18^

The time and length scales covered by QENS perfectly overlap with those typically probed by classical MD simulation.^19^ This correspondence makes MD simulations a powerful complementary tool for interpreting QENS data, enabling the computation of a “noise-free” dynamic structure factor directly from the simulation trajectory.^20–23^ A perfectly accurate MD simulation could be directly compared with observed QENS measurements to provide atomistic insights that cannot be obtained from experiments alone. However, the interatomic potentials used in classical MD simulations approximate atomic and molecular interactions and, therefore, cannot achieve chemical accuracy. Nevertheless, classical simulations can still be used to inform the analysis of QENS measurements in a range of different ways,^10,24^ including the use of real-space analysis of molecular dynamics simulations as a qualitative model of the dynamics.

The incoherent and total dynamic structure factors were calculated from MD simulations of benzene at 290 K, using MDANSE^23^ (full simulation details in section SI.I of the Supporting Information). Figure 1 compares the experimental QENS data (collected at the IRIS instrument, ISIS Neutron and Muon Source,^25^ using the PG 002 analyzer crystal, giving dynamic ranges for *Q* of 0.5–1.8 Å^–1^ and for ω of ±0.4 meV (see section SI.II for further experimental details)). We present the incoherent-only and total dynamic structure factors, as characterized by the elastic peak integral and the reduced second spectral moment, respectively, as descriptors of liquid structure and dynamics.^26^ Although the simulations generally capture the structure and dynamics, there is a discrepancy between the experimental and incoherent-only simulation data. This feature is on the length scale of a single benzene molecule, i.e., *Q* ≈ 1.3 Å^–1^ corresponding to around 4.8 Å, and is indicative of de Gennes narrowing (see Figure SI.1), which has been observed previously in the coherent dynamic structure factor.^27,28^ This anomaly indicates that even for hydrogenous benzene, the common approach to overlook coherent scattering is not suitable,^8^ necessitating either more complex fitting models that represent coherent scattering or making use of p-QENS, which allows the coherent and incoherent dynamic structure factors to be separated.^29^

**Figure 1:**
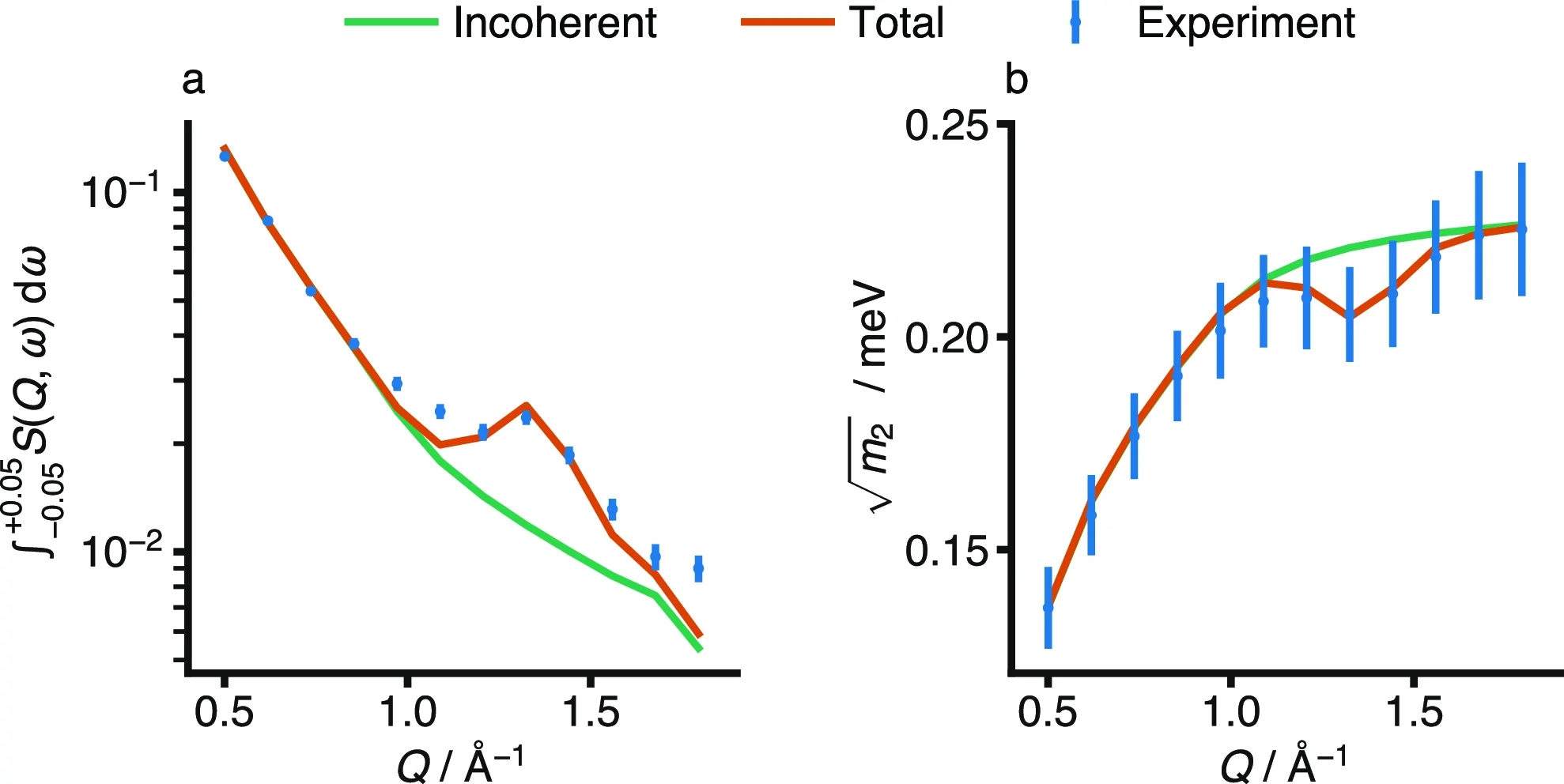
Comparison of the structure and dynamics of experimental (blue; error bars indicate a single standard deviation) and simulated (total dynamic structure factors (orange) and incoherent-only (green)) benzene: (a) a ±0.05 meV peak integral of the QENS spectra and (b) the reduced second spectral moment.

To choose the appropriate analytical model for interpreting the QENS data, we can analyze components of the molecular dynamics simulations, which provide a real-space description of molecular motion. First, we consider translational motion, characterized by the mean-squared displacement (Figure 2a), which shows a single linear regime, indicating the presence of a single translational mode with a self-diffusion coefficient of 0.142 ± 0.002 Å^2^ ps^–1^ (see section SI.IV for details).^30^ While benzene has been shown to hop between transient cages,^31^ these hops are too fast to be observed by QENS measurements. In the QENS data, a single translational mode would present itself as an ω-dependent Lorentzian function, the width of which will increase linearly as a function of *Q* ^2^, i.e., the Fickian diffusion QENS model.^3^

**Figure 2:**
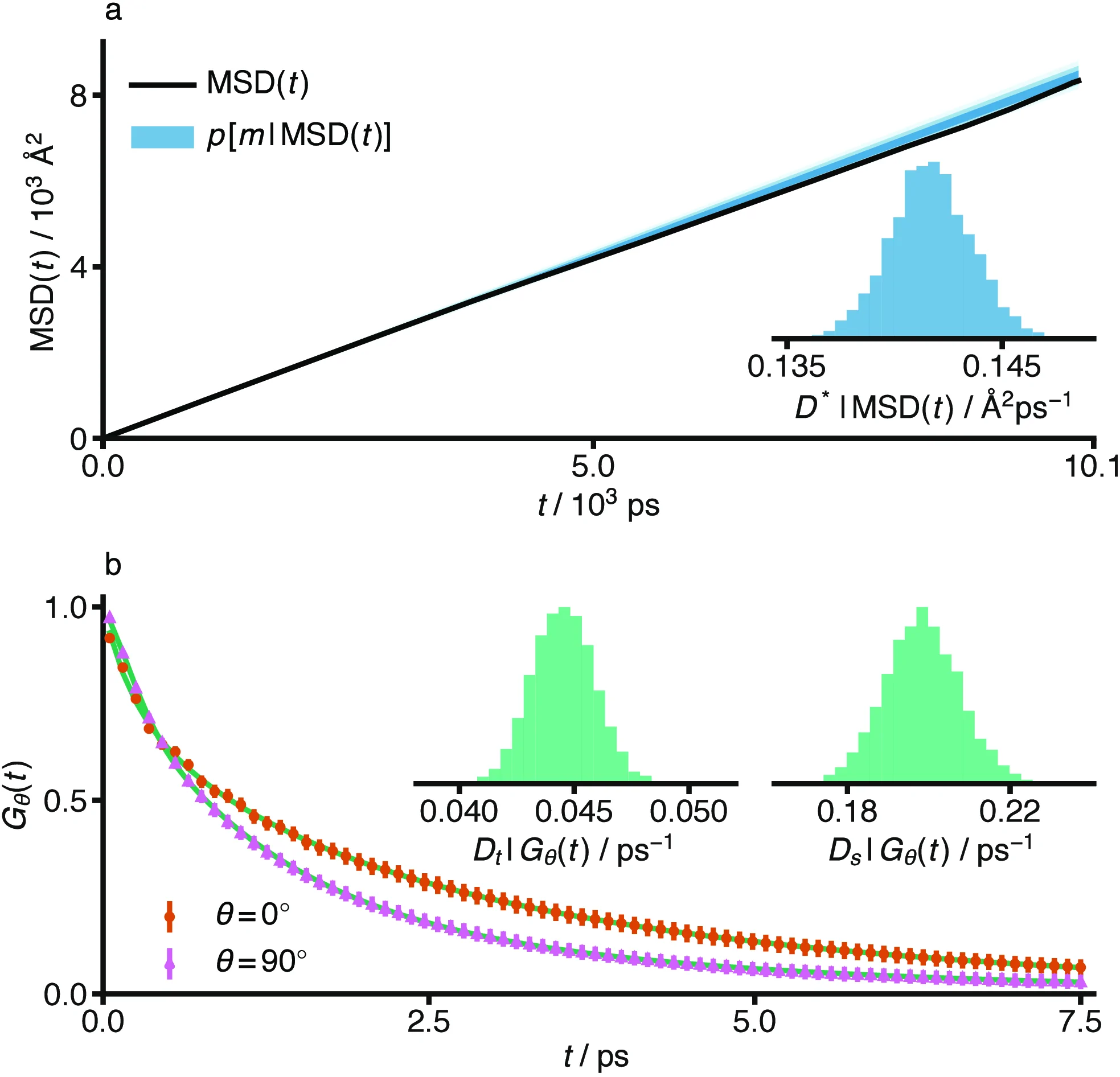
Real-space analysis of dynamics from simulation. (a) Observed mean-squared displacement (black line) with posterior distribution of linear models (blue shading indicating 1σ, 2σ, and 3σ credible intervals). The inset shows the marginal posterior distribution of self-diffusion coefficients from the linear models in panel a. (b) Rotational autocorrelation function of vectors parallel (θ = 0°; orange dots with error bars indicating a single standard deviation) and perpendicular (θ = 90°, pink triangles) to the benzene principal axis of rotation, and maximum *a posteriori* fitted models (solid green lines). The inset shows the marginal posterior distributions of the rotational diffusion coefficients found by sampling eqs 1 and 2 given both rotational autocorrelation functions.

Next, we consider rotational motion from the MD simulations. The benzene molecule is an oblate symmetric top with D_6*h*_ symmetry. It has been observed from model fitting to MD simulation and NMR and Raman spectroscopy data that the spinning of the benzene molecule around its principal axis is 2.3 ± 0.9 ^32^ times faster than the tumbling through the plane of the molecule.^33^ However, previous QENS measurements were unable to discriminate between the spinning and tumbling motions.^18^

To probe the rotational anisotropy in our simulation, the rotational autocorrelation functions for vectors parallel and perpendicular to the principal axis of rotation were calculated (Figure 2b). The perpendicular rotational autocorrelation function decays significantly faster than the parallel one, as the former is associated with both fast spinning and tumbling. In contrast, the latter is influenced by tumbling only. For an anisotropic axially symmetric rotor, like benzene, as the autocorrelation time, *t*, varies, the perpendicular rotational autocorrelation function will follow

$$G_{90{}^{\circ}}\left(t\right)=\frac{1}{4}\;\exp\left(-6D_{t}t\right)+\frac{3}{4}\;\exp\left[-\left(2D_{t}+4D_{s}\right)t\right]$$ 1

and the parallel autocorrelation time is described by

$$G_{0{}^{\circ}}\left(t\right)=\exp\left(-6D_{t}t\right)$$ 2

where *D*_t_ and *D*_s_ are the tumbling and spinning diffusion coefficients, respectively (see section SI.V for the full derivation). We use these models, along with additional functional descriptions of fast vibrations and librations (full details of this analysis are given in section SI.IV), to simultaneously estimate the posterior distributions for *D*_t_ and *D*_s_ (Figure 2b, inset). The difference between these two diffusion coefficients is around 0.15 ps^–1^, which is in principle within the temporal range of many QENS instruments. Notably, we find a larger anisotropy, of around 4.5, than in previous experimental and simulation studies. However, the model fitting approach used previously is inconsistent. A single-exponential function is used to infer a single effective time scale; this fails to model higher-energy local motions and artificially increases *D*_t_ (discussed in detail in section SI.VII), leading to an underestimation of the rotational anisotropy of benzene.

To assess whether the rotational anisotropy of benzene can be observed in QENS measurements, an appropriate analytical model for the dynamic structure factor must be used. To the best of our knowledge, no such model has been applied previously; hence, it is necessary to derive a *Q*/ω-dependent analytical model for the anisotropic rotation of an axially symmetric rotor. This derivation is given in section SI.V and when expanded to the second-order spherical Bessel functions is

$$S_{R}\left(Q,\omega \right)={j_{0}}^{2}\left(QR\right)\delta \left(\omega \right)+3{j_{1}}^{2}\left(QR\right)\left(\frac{1}{\pi }\frac{\Gamma_{1}}{\omega^{2}+{\Gamma_{1}}^{2}}\right)+5{j_{2}}^{2}\left(QR\right)\left(\frac{1}{4}\frac{1}{\pi }\frac{\Gamma_{2}}{\omega^{2}+{\Gamma_{2}}^{2}}+\frac{3}{4}\frac{1}{\pi }\frac{\Gamma_{3}}{\omega^{2}+{\Gamma_{3}}^{2}}\right)$$ 3

where *j*_*n*_(*QR*) represents the *n*th order spherical Bessel function with a radius of gyration *R*, which was fixed to 2.48 Å for benzene, and δ­(ω) is a Dirac delta function. The individual Γ values consist of the spinning and tumbling rotational diffusion coefficients as follows: Γ_1_ = *D*_s_ + *D*_t_, Γ_2_ = 6*D*_t_, and Γ_3_ = 4*D*_s_ + 2*D*_t_.

The real-space analysis of the simulation suggests that QENS may be used to distinguish between the two rotational modes of benzene. If the rates of the two rotations are too similar to discern with QENS, there is no advantage to the more complex anisotropic model in eq 3 over the simpler isotropic rotation model (eq SI.32).^34^ To determine which model better represents the observed data, we turn to Bayesian model selection, which allows models with different numbers of fitted parameters to be compared, penalizing more complex models when they are unnecessary.^14,35,36^

Bayesian model selection involves estimating the Bayesian evidence for some model, *m*

p(m|D)∝p(D|m)p(m) 4

where *p*(*m*) is a prior probability associated with the model and *p*(**D** | *m*) is the likelihood that the data, **D**, would be observed given the model, regardless of the model parameters, **θ**. This likelihood is found as the integral over the posterior parameter volume

$$p\left(D|m\right)=\int ...\int_{\theta_{m}}p\left(D\;|\;\theta_{m},m\right)p\left(\theta_{m}\;|\;m\right)\;d\theta_{m}$$ 5

Each additional parameter in an analytical model increases the dimensionality of the integral, leading to an exponential increase in the effective prior volume. Therefore, if the added parameter is not informative, the region of high likelihood occupies a much smaller fraction of that volume, thereby reducing the integral and, consequently, the Bayesian evidence. For there to be “strong evidence” for a more complex model, *m*_1_ (i.e., the anisotropic model, with more parameters), the natural logarithm of this model’s evidence should be at least 5 greater than for the simpler model, *m*_0_, given the same data, i.e., ln­[*p*(*m*_1_ | **D**)] ≥ ln­[*p*(*m*_0_ | **D**)] + 5.^37^

To assess whether our QENS measurement can distinguish between the two rotational modes, we first consider a simulated dynamic structure factor that contains only rotational information. We achieve this by centring each benzene molecule in the calculated simulation trajectory at its initial molecular center of mass at each simulation time step, thereby removing translational motion from the trajectory (details are provided in section SI.VIII). From this trajectory, we calculate the incoherent dynamic structure factor with a symmetric ω-dynamic range of ±0.4 meV, matching that of the experimental data collected at IRIS and assuming Poisson counting statistics to estimate the uncertainty. Given this simulated signal, the Bayesian evidence for each analytical model was estimated using nested sampling,^38^ with the prior probability for each model set to 1. Comparing this evidence showed strong support for the more complex anisotropic model, with a log-Bayesian evidence value of −56.0, compared to −78.2 for the isotropic model.

Nested sampling simultaneously estimates the posterior distribution of the parameters; the marginal posterior distributions for the two rotational diffusion coefficients are shown in Figure 3. These distributions are compared with the mean values of the rotational diffusion coefficients obtained directly from the simulation’s rotational autocorrelation function. The posterior distribution of eq 3 gives a larger value for the rotational diffusion coefficient of spinning when compared with those from the rotational autocorrelation function, likely arising from the difficulty of empirically separating motions within the incoherent dynamic structure factor, even for the deconvolved rotational model.

**Figure 3:**
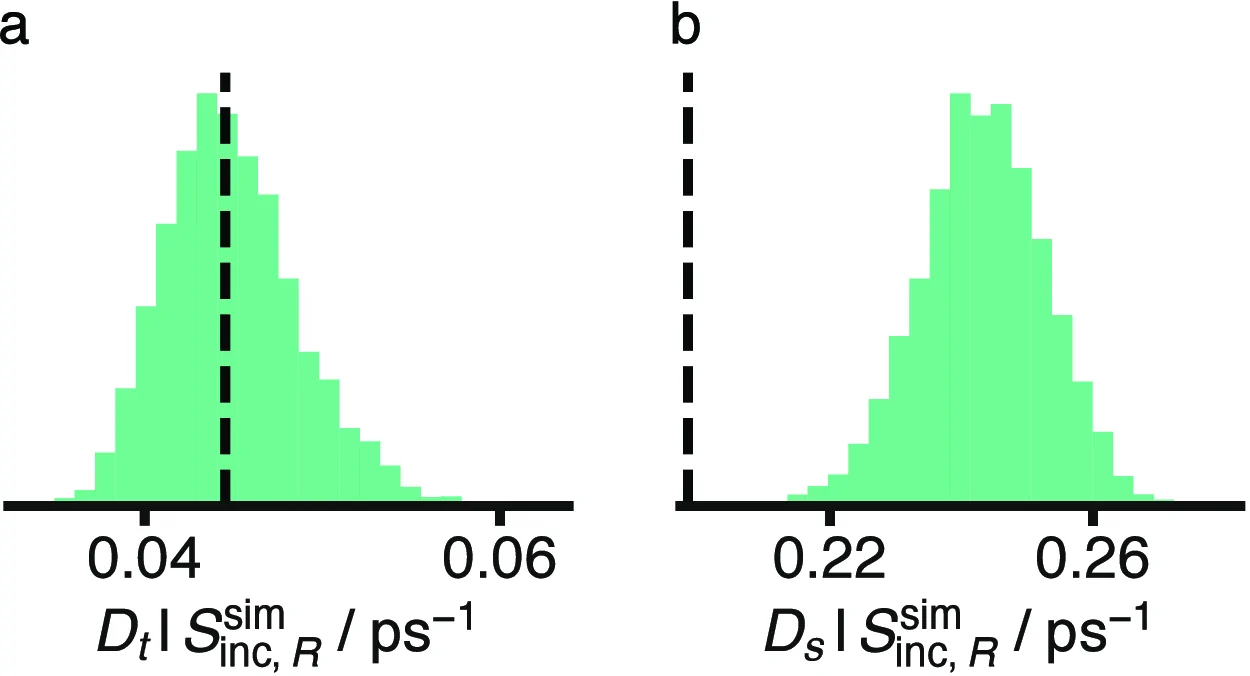
Marginal posterior distributions of the (a) tumbling and (b) spinning diffusion coefficients for eq 3 for the incoherent dynamic structure factor computed from the rotation-only simulation. Vertical black lines show the mean of the marginal posterior distributions from the rotational autocorrelation function.

Having shown that, in the hypothetical case where only rotation is present in the incoherent dynamic structure factor, one can observe individual rotations, we now consider whether the anisotropic model retains strong support when translational motion is also present. Therefore, we convolve our model for rotation with one for Fickian diffusion at each *Q* value

$$S_{\mathrm{inc}}\left(Q,\omega \right)=e^{-Q^{2}\left\langle u^{2}\right\rangle /3}\left[\frac{1}{\pi }\frac{D^{*}Q^{2}}{\omega^{2}+{\left(D^{*}Q^{2}\right)}^{*2}}\right]⊗S_{R}\left(Q,\omega \right)$$ 6

where D* is the Fickian translational self-diffusion coefficient, ⟨*u* ^2^⟩ is the mean-squared displacement for the Debye–Waller factor, and *S*_R_(*Q*, ω) is either eq 3 or the isotropic rotational model (eq SI.32). Equation 6 is significantly more constrained, using the isotropic or anisotropic models, than the traditional approach to QENS analysis outlined in the introduction.

Figure 4a presents the difference in Bayesian evidence between the isotropic and anisotropic models, using the incoherent dynamic structure factor from the full simulation trajectory. Over a small ω-dynamic range of ±0.5 meV, slightly greater than that of the IRIS measurements, it is not possible to distinguish between the two models. However, as the ω-dynamic range is increased, the Bayesian evidence for the more complex model increases, and from ±0.75 to ±1.25 meV, there is strong evidence for the anisotropic model. Representative marginal posterior distributions of the translational self-diffusion coefficient and rotational diffusion coefficients are given in panels b–d of Figure 4 at ±1.25 meV. While the two rotational diffusion coefficients are distinct, their values differ slightly from those found by analyzing the decomposed simulation. This difference may be due to the assumption that translation and rotation are completely uncorrelated, which is not accounted for when the molecular translation is removed, or the use of the numerical convolution to evaluate eq 6. Over larger ω-dynamic ranges, high-energy local motions that were observed in the real-space analysis above are present in the simulated QENS signal; this was also characterized experimentally previously.^18^ Neither eq 6 nor the isotropic model describes these high-energy motions; therefore, the Bayesian evidence suggests that we should accept the “null hypothesis” of the less complex model.

**Figure 4:**
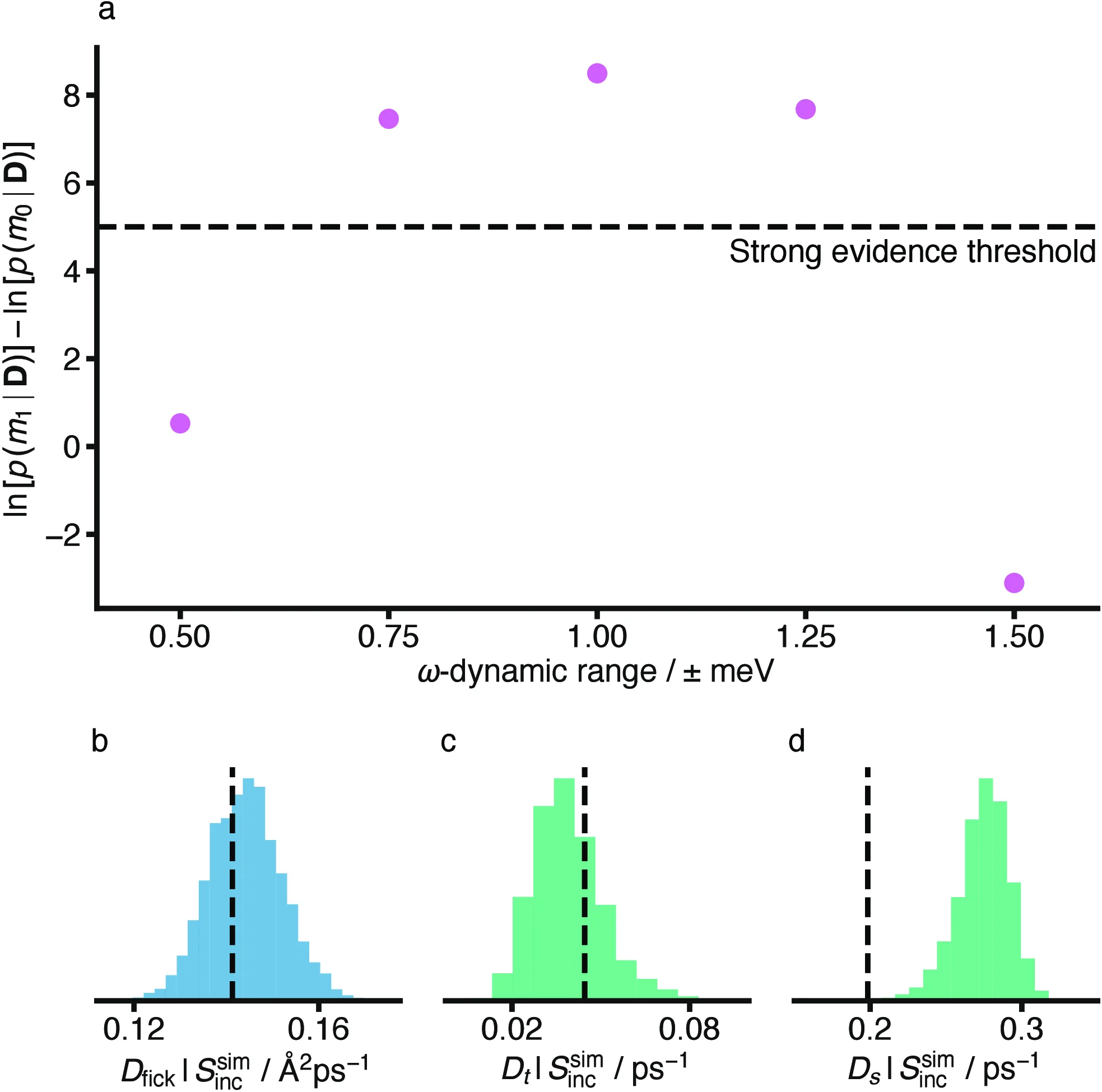
Analysis of the ω-dynamic range dependence of Bayesian evidence using QENS from the full simulation. (a) Bayesian evidence for the anisotropic model compared with the isotropic model as a function of ω-dynamic range, where the threshold for strong evidence is indicated with a dashed black line. (b) Fickian self-diffusion, (c) tumbling rotational, and (d) spinning rotational diffusion coefficients marginal posterior distributions, obtained for the anisotropic model, given the simulated incoherent QENS signal at an ω-dynamic range of ±1.25 meV, compared with the mean of the distributions obtained directly from simulation trajectory (dashed vertical black lines).

We have shown that to observe rotational anisotropy in benzene from a simulated QENS signal, it is necessary to have an ω-dynamic range of ±0.75 to ±1.25 meV and to minimize the presence of coherent scattering. This dynamic range exceeds that of the IRIS instrument with the PG 002 analyzer crystal, and as shown in Figure 1, it is not possible to remove coherent scattering from this signal. Therefore, we measured the QENS at the LET instrument (ISIS Neutron and Muon Source) in the polarization analysis configuration using incident energies of 1.97 and 3.60 meV; these data were rebinned and truncated to give an ω-dynamic range of ±1.25 meV.^29^ We chose the largest ω-dynamic range to account for the possibility of faster spinning than was observed in simulation. Polarization analysis allows for the decomposition of the total dynamic structure factor into its individual components; this means that the Bayesian evidence for the two analysis models can be found given the purely incoherent data. There was strong support for the more complex anisotropic model, with a log-Bayesian evidence value of −2874.3, compared to a value of −3032.8 for the isotropic model, given the noisy experimental data. Figure 5 compares the *Q*-dependent anisotropic model with the measured LET data at each incident energy, showing strong agreement between the two (this is further compared with the lower-evidence isotropic model in Figures SI.7 and SI.8); the ratio between the model and the experiment shows noise, with no structuring present in the error (see section SI.IX for analysis details).

**Figure 5:**
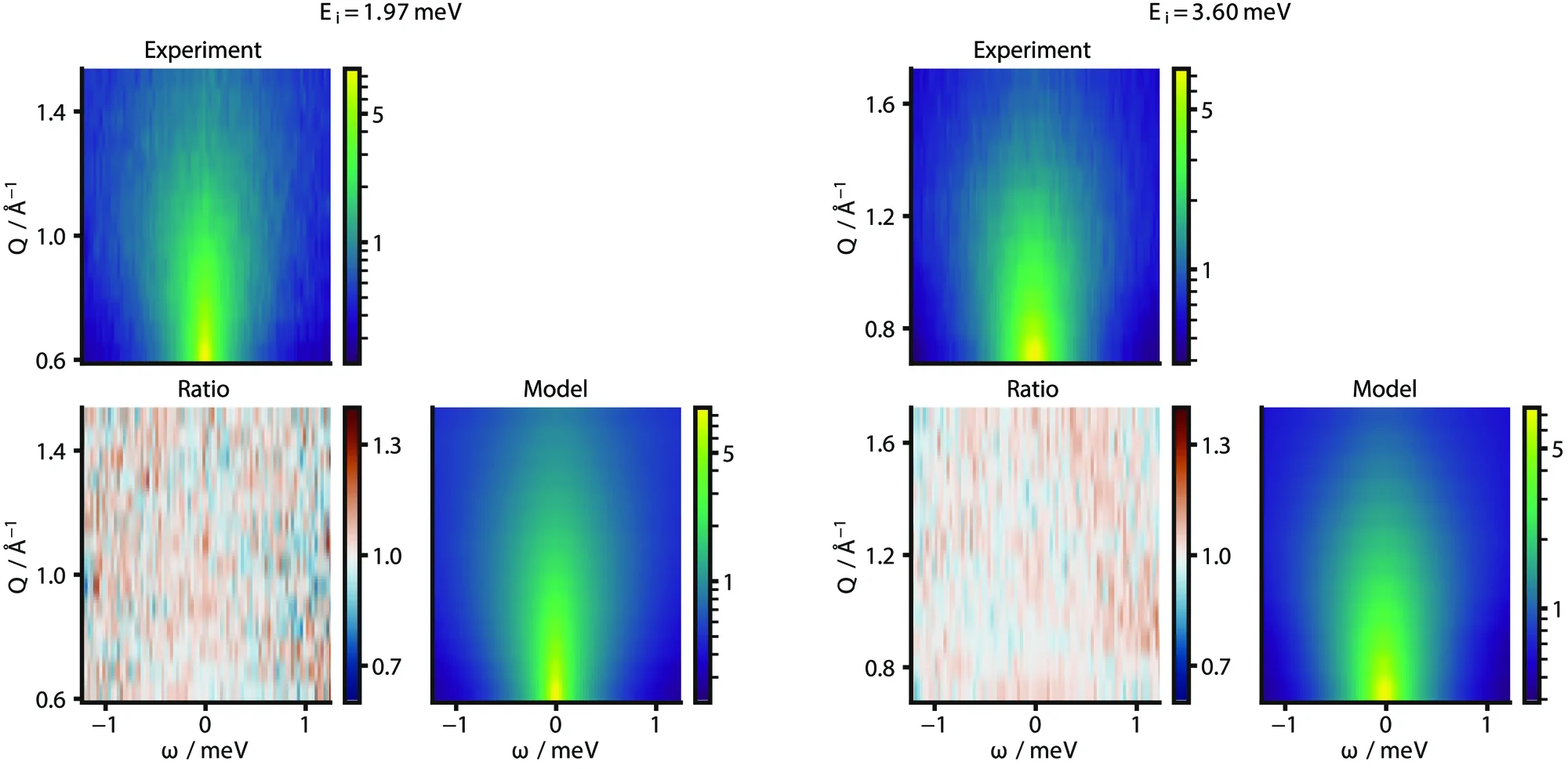
Anisotropic model maximum *a posteriori* given experimental LET data at incident energies of 1.97 and 3.60 meV. Comparison of the incoherent dynamic structure factor from the experimental data, corefined anisotropic model, and their ratio. Individual *Q*-dependent line fits available in Figures SI.7 and SI.8.

Given the LET data, the full posterior distribution for the anisotropic analytical model was also found. All three experimental diffusion coefficients differ slightly from those obtained from the MD simulation, with the experiment showing faster Fickian diffusion and spinning rotational diffusion, and slower tumbling rotational diffusion. The marginal posterior distribution for the Fickian self-diffusion coefficient is shown in Figure 6a, with a mean and 1σ credible interval of 0.182 ± 0.002 Å^2^ ps^–1^. This value agrees well with previous QENS analysis that found the self-diffusion coefficient to be ∼0.16 Å^2^ ps^–1^ ^18^ and other experimental measurements, such as the value of 0.186 Å^2^ p^–1^ at 288.2 K.^39^

**Figure 6:**
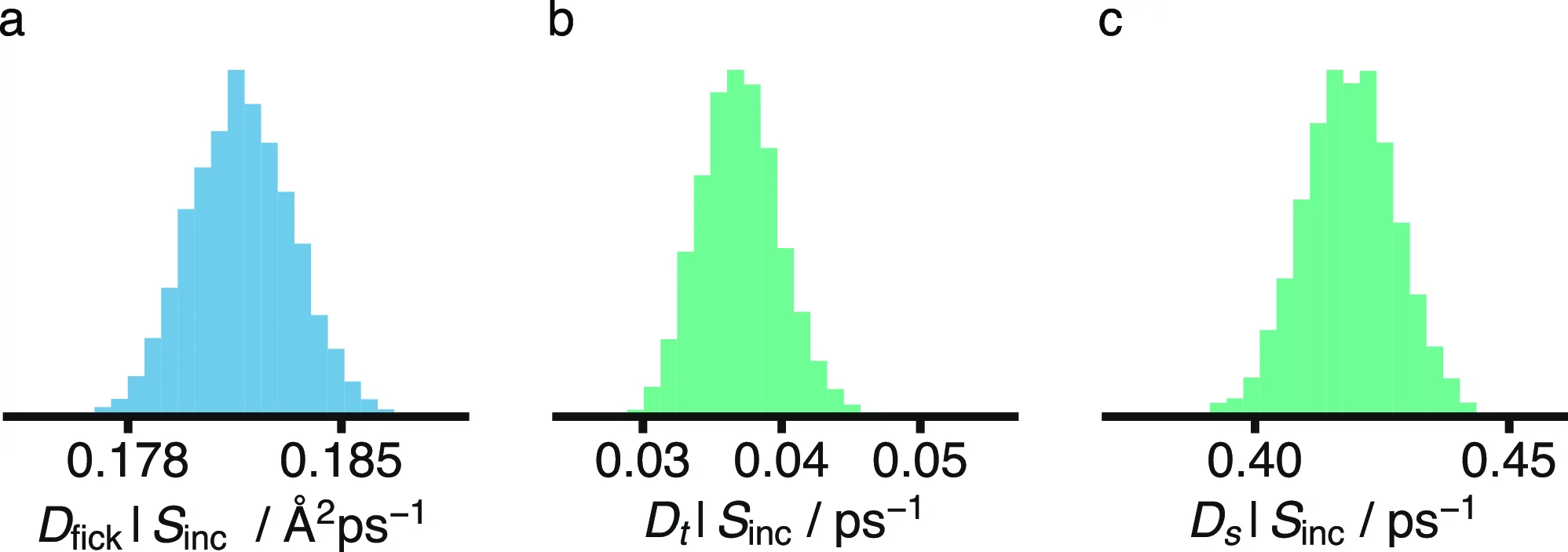
Marginal posterior distributions for the anisotropic model given data at both incident energies: (a) Fickian self-diffusion coefficient and (b) tumbling and (c) spinning rotational diffusion coefficients.

Significantly, the experimental estimates for the two rotational diffusion coefficients are distinct and non-zero (Figure 6b,c); by addressing the issues highlighted in the introduction, it is possible, for the first time, to distinguish the anisotropy of the benzene rotation with QENS. Previously, it was not possible to observe anisotropy, and a single rotational diffusion coefficient was obtained from QENS data.^18^ This single value closely matches the average of the tumbling and spinning diffusion coefficients determined in this work. The marginal posterior distributions for *D*_s_ and *D*_t_ indicate faster and slower rotation, respectively, than observed in our simulations, leading to an anisotropy ratio of around 11. This anisotropy ratio is greater than that found from simulation alone, which may be rationalized by recognizing that experimentally, liquid benzene has been shown to form “T”- and “Y”-shaped perpendicular dimers, as well as parallel π-stacked agglomerates,^40,41^ structures that are not observed in our simulations (see section SI.XI) and would make tumbling energetically unfavorable compared to spinning. Though interesting, this increased anisotropy requires further investigation to conclusively say it was underestimated in previous work. By using simulations as a quantitative model for the experimental data, it has been possible to observe anisotropy that could not be measured previously with QENS.

We have derived a detailed, fully *Q*-dependent analytical model for anisotropic rotational motion and explicitly evaluated its statistical sensitivity within the *Q* and ω ranges accessible to QENS, using Bayesian analysis informed by MD simulations. Furthermore, we employed Bayesian model selection to compare this model with simpler, lower-parameter descriptions, demonstrating that an anisotropic model provides a more accurate and physically nuanced account of the data. Importantly, analysis of the simulated ω sensitivity identified the regime in which the anisotropic model is statistically identifiable, guiding experimental design. Polarization-resolved QENS then isolated the incoherent scattering component, which is directly described by the analytical model, ensuring consistency between theoretical assumptions and measured observables. By integrating these elements, we have shown, for the first time, that QENS can fully resolve both translational and anisotropic rotational dynamics in liquid benzene, explicitly distinguishing two distinct rotational modes and suggesting that previous estimates of anisotropy in benzene may require reconsideration. More broadly, this enhanced resolution of translational and rotational motion has significant implications for catalysis and functional porous materials. Quantitative characterization of molecular diffusion and anisotropic rotation within zeolite catalysts provides direct measures of bonding constraints and local transport processes, which determine the catalytic rate and conversion efficiency. We hope that this work will inspire others to consider more carefully the information that may be drawn from their QENS data and enable other researchers to maximize the information obtained from this powerful technique.

## Supplementary Material



## Data Availability

Raw experimental QENS data are available from the ISIS Neutron and Muon Source Data Journal.^42,43^ The data used to generate the figures in this paper are available on Zenodo,^44^ and all of the code required to recreate them can be found as a showyourwork workflow in the appropriate GitHub repository,^45^ under MIT (code) and CC BY-SA 4.0 (figures and text) licenses. Also present in this repository is the custom Python code used to perform the analysis presented in this work, which is similarly fully computationally reproducible.

## Notes

- Descriptions of the simulation parameters, experimental measurements, and data reduction; details of the simulation translational motion analysis; a more in-depth discussion of the presence of de Gennes narrowing; mathematical derivations of the rotational autocorrelation function and dynamic structure factor for an oblate symmetric top; details of the fitting approach used for the rotational autocorrelation function analysis; comparison of the rotational autocorrelation function analysis used in the literature with that used in this work; information about the QENS analysis from the decomposed simulation trajectory; details of the QENS model fitting presented in the text; figures showing the QENS signal for each *Q* bin compared with the maximum *a posteriori* anisotropic model at the same *Q* bin; and position and orientation probability density function for the benzene simulation (PDF)
