# Supplementary material for: Lost in Translation: Simulation-Informed Bayesian Inference Improves Characterization of Molecular Motion from Neutron Scattering
Source: J Phys Chem Lett. 2026 Jul 9;17(29):8411–8. doi: 10.1021/acs.jpclett.6c01399 (PMC13557505; doi:10.1021/acs.jpclett.6c01399)
Supplement: Supplementary file 1 [file jz6c01399_si_001.pdf]

Supplemental Material for "Lost in Translation: Simulation-Informed Bayesian Inference Improves Characterisation of Molecular Motion From Neutron Scattering"

Harry Richardson,[1] Kit McColl,[2,3] Gøran J. Nilsen,[4,5] Jeff Armstrong,[4,6,*] and Andrew R. McCluskey[1,7,†]

[1] *Centre for Computational Chemistry, School of Chemistry, University of Bristol, Cantock's Close, Bristol, BS8 1TS, UK.*
[2] *Department of Materials, University of Oxford, Parks Road, Oxford OX1 3PH, UK.*
[3] *The Faraday Institution, Quad One, Harwell Science and Innovation Campus, Didcot, OX11 0RA, UK.*
[4] *ISIS Pulsed Neutron and Muon Facility, Rutherford Appleton Laboratory, Didcot, OX11 0QX, UK.*
[5] *Department of Mathematics and Physics, University of Stavanger, 4036 Stavanger, NO.*
[6] *Department of Chemistry, University of Bath, Claverton Down, Bath, BA2 7AY, UK.*
[7] *Diamond Light Source, Harwell Campus, Didcot, OX11 0DE, UK.*

SI.I: Classical Molecular Dynamics Simulation Parameters

Molecular dynamics simulations were carried out in LAMMPS [1]. Initial configurations of the bulk liquid were constructed by the $9 \times 9 \times 9$ replication of a single molecule (resulting in 729 molecules) generated using LigParGen [2]. All atomic charges and intra- and intermolecular interactions were modelled using the OPLS-AA force field [3]. Lennard-Jones and real-space Coulombic interactions were truncated at 9 Å, with long-range electrostatics evaluated using the particle-particle particle-mesh (PPPM) method [4]. The system was equilibrated for 20 ps at 10 K with a 1 fs timestep using the NVT ensemble and a Nose-Hoover thermostat [5–7], with a temperature damping parameter of 100 fs. The temperature of the system was then ramped from (10 to 290) K at 1 atm in the NPT ensemble using a Nose/Hoover temperature thermostat and Nose/Hoover pressure barostat over 1 ns [8], with the same temperature damping as previously and a pressure damping parameter of 1000 fs. Following the temperature ramp, the system was run at 290 K and 1 atm for 0.5 ns and the trajectory frame reflecting the mean box dimensions was taken, resulting in a 47.7 Å cubic simulation cell and simulation density of $0.869\,\mathrm{g\,cm^{-3}}$ (compared with an experimental density of $0.879\,\mathrm{g\,cm^{-3}}$ for liquid benzene at 293.15 K [9]). The production trajectory was generated from a 10 ns NVT ensemble run at 290 K, starting from the selected frame, with frames stored every 0.5 ps. For the rotational autocorrelation functions discussed in the main text, the same equilibration and system size were used, but a production trajectory of 1 ns sampled every 0.05 ps was generated. The autocorrelation functions were then calculated over 100 independent 10 ps windows within the trajectory.

SI.II: Quasi-Elastic Neutron Scattering

1. Sample and Measurement

On IRIS, neat benzene (Sigma-Aldrich, 99.8 % purity) was loaded into aluminium sample cans, with a 0.25 mm thick annular space, in a fume hood to minimise exposure to moisture in the air, and the cans were sealed with indium wire. The sample was loaded onto the IRIS instrument and measured for 190 min at 290 K. An empty aluminium can was used as a background, and a previously measured vanadium sample served as the resolution function.

On LET, the same benzene was loaded into a 15 mm (outer diameter) aluminium can with a 0.25 mm thick annular space. The can was sealed with indium wire, and then loaded into an Orange cryostat on LET, where it was measured for around 8 h (480 min) at 290 K and 2 h at 260 K. A total of 4 incident energies, E_i, were measured using repetition rate multiplication: (8.61, 3.60, 1.95, and 1.23) meV. The energy resolutions at these energies were 300 µeV, 85 µeV, 45 µeV and 15 µeV and the maximum accessible energy dynamic ranges were ±6.88 meV, ±2.88 meV, ±1.576 meV and ±0.992 meV respectively. The two intermediate incident energies were used for the analysis, as they provide sufficient resolution and dynamic range to capture the expected dynamics in benzene. An empty can was used as a background, and a frozen benzene sample at 260 K was measured and used for the resolution function (see detailed discussion of data reduction below).

Polarisation analysis on LET was achieved using a polarised neutron beam, from an $m = 5$ Fe/Si supermirror V-cavity, and the scattered polarisation was analysed with a hyperpolarised ^{3}He spin-filter cell. A current-ramped precession coil π-flipper is placed after the polariser to measure the spin-flip and non-spin flip intensities, S_{sf} and S_{nsf}, as a function of scattering angle, θ, and energy transfer, ω. Measurements of the time-dependent polarisation

* jeff.armstrong@stfc.ac.uk
† andrew.mccluskey@bristol.ac.uk

efficiency of the instrument taken using a monitor in the direct beam were used to correct the raw spin-flip and non-spin-flip intensities according to the procedure described in [10, 11], following which they were converted to dynamic structure factors, $S_{\mathrm{sf}}(Q,\omega)$ and $S_{\mathrm{nsf}}(Q,\omega)$. The spin-incoherent dynamic structure factor S_{inc} was finally obtained from the corrected spin flip dynamic structure factor according to:

$$S_{\mathrm{inc}}(Q,\omega) = \frac{3}{2} S_{\mathrm{sf}}(Q,\omega). \tag{SI.1}$$

2. Data Reduction

The experimental IRIS data was binned into 12 Q-bins, each with a width of $0.113\,\text{Å}^{-1}$ across the 51 detectors with a total Q-range of $(0.44 \text{ to } 1.79)\,\text{Å}^{-1}$. A vanadium sample was used to determine the instrument's resolution function. The same sample was used to correct for detector efficiency, by normalising each QENS spectrum with the energy-integrated vanadium sample intensity at the corresponding Q-value. Data measured from an empty sample can were used to quantify the instrument background and were subtracted from both the benzene and vanadium data.

The experimental p-LET data were initially binned into 30 Q-bins for each incident energy. This data was then sampled in the chosen Q range, based on the region of interest and instrumental constraints $(0.67 \text{ to } 1.72)\,\text{Å}^{-1}$, for $E_i = 3.60\,\text{meV}$ and between $(0.59 \text{ to } 1.53)\,\text{Å}^{-1}$ for $E_i = 1.97\,\text{meV}$. This resulted in 12 and 14 bins of width $0.096\,\text{Å}^{-1}$ and $0.073\,\text{Å}^{-1}$ for 3.6 meV and 1.97 meV, respectively, within the chosen Q-range. Variations in instrumental detector efficiency were corrected using a "white-beam" vanadium measurement collected at the beginning of the cycle. A frozen benzene measurement at 260 K was used as an approximation of the instrumental resolution function. This frozen sample is not a perfect representation of the true resolution, as some molecular mobility remains at this temperature. Although vanadium is often used to estimate instrumental resolution, its scattering profile differs from that of the sample, particularly in the high-energy-transfer tails, which can introduce systematic distortions in the convolution during the final model.

SI.III: Evidence of de Gennes Narrowing

We compare the dynamic structure factor peak integral and reduced second spectral moment of the incoherent and total dynamic structure factor in Fig. 1 of the main text and attribute the discrepancy between these to de Gennes narrowing [12]. Strictly, de Gennes narrowing is the narrowing of the coherent dynamic structure factor at wavevectors corresponding to peaks in the static structure factor. To show that what is observed here is indeed de Gennes narrowing, in Fig. SI.1 we include the coherent contribution in the reduced second spectral moment plot from Fig. 1b, which shows the depression of the coherent signal, indicative of de Gennes narrowing.

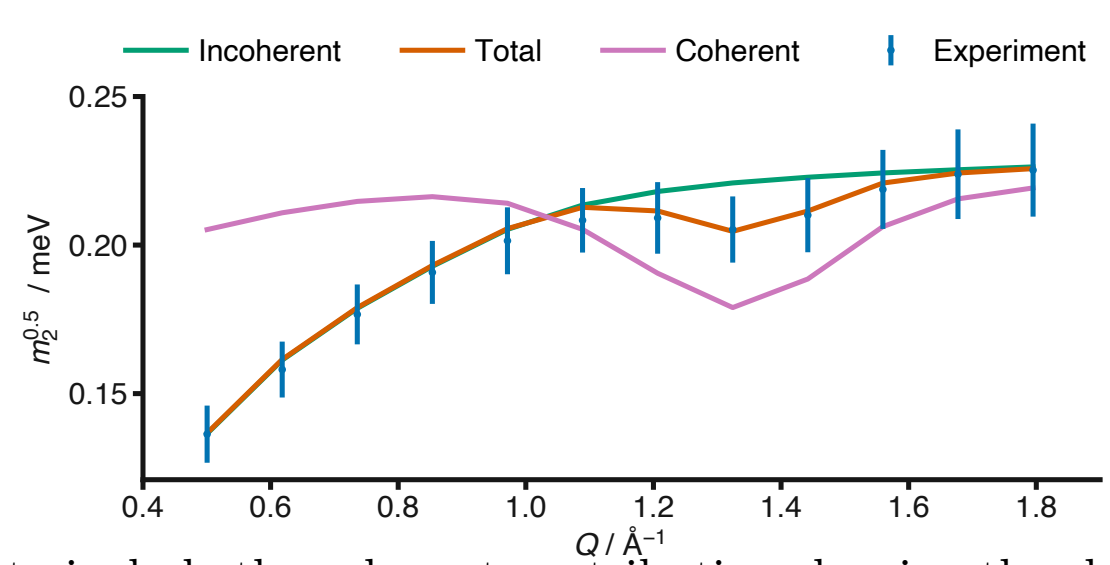

FIG. SI.1. An adaptation of Fig. 1b to include the coherent contribution showing the characteristic depression of the coherent reduced second moment that is indicative of de Gennes narrowing.

SI.IV: Translation Diffusion Analysis

The translational diffusion analysis made use of the KINISI software [13]. The molecular dynamics trajectory was subsampled at every 5 ps and the centre-of-mass functionality [14] was used to find the benzene mean-squared displacements at lag times of (5 to 10 000) ps in intervals of 5 ps. The Bayesian regression scheme implemented in KINISI was then used to estimate the self-diffusion coefficient [15], with the start of the diffusion regime fitting window at 500 ps.

SI.V: Mathematical Derivations of Models

1. Rotational Autocorrelation Function

We start by deriving the rank-l correlation function for an anisotropically axially symmetric rotator. Specifically, this considers the oblate symmetric top benzene. The Wigner D-functions, which form an orthonormal basis set for the 3D rotation group, SO(3), have the following eigenvalues [16],

$$\lambda_{lm} = D_t l(l+1) + m^2(D_s - D_t), \tag{SI.2}$$

where D_t and D_s are the rotational diffusion coefficients for the benzene tumbling and spinning, respectively, l is the magnitude of the angular momentum vector, and m is the projection of that vector [17]. The rank-l correlation function, $C_l(t,\theta)$ is then defined as,

$$C_l(t,\theta) = \sum_{m=-l}^{l} [d^l_{m0}(\theta)]^2 \exp\left(-\lambda_{lm} t\right), \tag{SI.3}$$

where d^l_{m0} is the appropriate element from the Wigner small-d matrix [16, 18].

Using Eq. SI.3, it is then possible to find any rank correlation function. For example, in the main text, we discuss the rotational autocorrelation function, $G_\theta(t)$, which follows the rank-2 correlation function,

$$C_2(t,\theta) = \frac{1}{4}\left(3\sin^2(\theta) - 2\right)^2 \exp\left[-t6D_t\right] + 12\exp\left[-t(4D_s + 2D_t)\right]\sin^4\left(\frac{\theta}{2}\right)\cos^4\left(\frac{\theta}{2}\right) + \frac{3}{4}\exp\left[-t(D_s + 5D_t)\right]\sin^2(2\theta). \tag{SI.4}$$

This result is equivalent to the Woessner equation used in previous NMR analysis of benzene dynamics [19, 20]. From this, the functions that describe the rotational autocorrelation functions for $\theta = 0°$ and $\theta = 90°$, i.e., parallel and perpendicular to the principal axis of rotation, can be obtained. These are

$$C_2(t, 0°) \equiv G_{0°}(t) = \exp\left[-t6D_t\right], \tag{SI.5}$$

and

$$C_2(t, 90°) \equiv G_{90°}(t) = \frac{3}{4}\exp\left[-t\left(4D_s + 2D_t\right)\right] + \frac{1}{4}\exp\left(-t6D_t\right). \tag{SI.6}$$

These can be rationalised, as the vector parallel ($\theta = 0$) to the principal axis is unaffected by the spinning motion, and hence contains only D_t. Meanwhile, the perpendicular vector is affected by both spinning and tumbling.

2. Dynamic Structure Factor

Now, we turn our focus to the derivation of the dynamic structure factor. We consider a rigid molecule with a single scattering nucleus located at a fixed distance R from the molecular centre. Its position in the laboratory frame is,

$$\mathbf{r}(t) = R\hat{\mathbf{u}}(t). \tag{SI.7}$$

where the change in direction of the unit vector $\hat{\mathbf{u}}$ is being probed. The intermediate scattering function for incoherent scattering is defined as,

$$I(Q,t) = \left\langle \exp\left(i\mathbf{Q}\cdot[\mathbf{r}(t) - \mathbf{r}(0)]\right)\right\rangle, \tag{SI.8}$$

where $\langle \ldots \rangle$ denotes an ensemble (and for a liquid, orientational) average. Substituting $\mathbf{r}(t) = R\hat{\mathbf{u}}(t)$ and $\mathbf{Q} = \hat{\mathbf{Q}}Q$ into the above gives,

$$I(Q,t) = \left\langle \exp\left(iQR\hat{\mathbf{Q}}\cdot\hat{\mathbf{u}}(t)\right)\exp\left(-iQR\hat{\mathbf{Q}}\cdot\hat{\mathbf{u}}(0)\right)\right\rangle, \tag{SI.9}$$

and since the intermediate scattering function is independent of $\hat{\mathbf{Q}}$, we can average over $\hat{\mathbf{Q}}$,

$$I(Q,t) = \left\langle \frac{1}{4\pi}\int \exp\left(iQR\hat{\mathbf{Q}}\cdot\hat{\mathbf{u}}(t)\right)\exp\left(-iQR\hat{\mathbf{Q}}\cdot\hat{\mathbf{u}}(0)\right)\mathrm{d}\Omega\right\rangle \tag{SI.10}$$

where Ω is the solid angle.

We can use the standard Rayleigh expansion to reframe these exponential forms using spherical harmonics, i.e., Y_{lm}, and spherical Bessel functions, $j_l(x)$,

$$\exp\left(i\mathbf{k}\cdot\mathbf{r}\right) = 4\pi\sum_{l=0}^{\infty} i^l j_l(kr) \sum_{m=-l}^{l} Y_{lm}^*(\hat{\mathbf{k}})Y_{lm}(\hat{\mathbf{r}}). \tag{SI.11}$$

Therefore, it is possible to rewrite the exponential functions in Eq. SI.10 as

$$\exp\left(iQR\hat{\mathbf{Q}}\cdot\hat{\mathbf{u}}(t)\right) = 4\pi\sum_{l=0}^{\infty} i^l j_l(QR) \sum_{m=-l}^{l} Y_{lm}^*(\hat{\mathbf{Q}})Y_{lm}\left[\hat{\mathbf{u}}(t)\right] \tag{SI.12}$$

and

$$\exp\left(-iQR\hat{\mathbf{Q}}\cdot\hat{\mathbf{u}}(0)\right) = 4\pi\sum_{l=0}^{\infty} i^l j_l(QR) \sum_{m=-l}^{l} Y_{lm}(\hat{\mathbf{Q}})Y_{lm}^*\left[\hat{\mathbf{u}}(0)\right]. \tag{SI.13}$$

At this stage, we observe that we will be able to take advantage of the orthogonality in the spherical harmonics containing $\hat{\mathbf{Q}}$,

$$\int Y_{lm}^*(\hat{\mathbf{Q}})Y_{l'm'}(\hat{\mathbf{Q}})\, \mathrm{d}\Omega_{\hat{\mathbf{Q}}} = \delta_{ll'}\delta_{mm'}. \tag{SI.14}$$

Using the orthogonality, we can integrate over $\hat{\mathbf{Q}}$ and use $i^l(-i) = 1$ to obtain

$$I(Q,t) = \left\langle 4\pi\sum_{l=0}^{\infty}\left[j_l(QR)\right]^2 \sum_{m=-l}^{l} Y_{lm}\left[\hat{\mathbf{u}}(t)\right] Y_{lm}^*\left[\hat{\mathbf{u}}(0)\right]\right\rangle. \tag{SI.15}$$

Next, we use the spherical harmonic addition theorem, which states

$$\sum_{m=-l}^{l} Y_{lm}\left(\hat{\mathbf{n}}\right) Y_{lm}^*\left(\hat{\mathbf{n}}'\right) = \frac{2l+1}{4\pi}P_l(\hat{\mathbf{n}}\cdot\hat{\mathbf{n}}'), \tag{SI.16}$$

where P_l is the Legende polynomial of order l, which is the definition of the rank-l orientational correlation function we derived earlier, i.e.,

$$C_l(t,\theta) = \left\langle P_l\left[\hat{\mathbf{u}}(t)\cdot\hat{\mathbf{u}}(0)\right]\right\rangle. \tag{SI.17}$$

Applying this identity leads to,

$$I(Q,t,\theta) = \sum_{l=0}^{\infty}(2l+1)\left[j_l(QR)\right]^2 C_l(t,\theta). \tag{SI.18}$$

From Eq. SI.18, we can find the intermediate scattering function for an anisotropic axially symmetric rotator, with a maximum l of 2, as,

$$\begin{aligned} I(Q,t,\theta) =& j_0(QR)^2 - 3j_1(QR)^2\left(\frac{1}{2}\exp\left[-t(D_s+D_t)\right]\cos\left(2\theta\right) + \frac{1}{2}\exp\left[-t(D_s+D_t)\right] + \exp\left(-t2D_t\right)\cos^2\left(\theta\right)\right) \\ &+ 5j_2(QR)^2\left(\frac{1}{4}\left(3\sin^2\left(\theta\right)-2\right)^2\exp\left(-t6D_t\right) + 12\exp\left[-t(4D_s+2D_t)\right]\sin^4\left(\frac{\theta}{2}\right)\cos^4\left(\frac{\theta}{2}\right)\right. \\ &\left.+ \frac{3}{4}\exp\left[-t(D_s+5D_t)\right]\sin^2\left(2\theta\right)\right). \end{aligned} \tag{SI.19}$$

This equation is still dependent on θ, the angle with respect to the principal rotational axis. At this stage, we observe that for benzene, all of the scattering nuclei sit in the plane of the molecule, corresponding to $\theta = \frac{\pi}{2}$. Therefore,

$$I(Q,t) = j_0(QR)^2 + 3j_1(QR)^2\exp\left[-t(D_s+D_t)\right] + 5j_2(QR)^2\left(\frac{3}{4}\exp\left[-t\left(4D_s+2D_t\right)\right] + \frac{1}{4}\exp\left(-t6D_t\right)\right). \tag{SI.20}$$

From the intermediate scattering functions, the dynamic structure factor can be found; a Fourier transform relates these, i.e.,

$$S(Q,\omega) = \frac{1}{2\pi}\int_{-\infty}^{\infty} e^{i\omega t} I(Q,t)\,\mathrm{d}t. \tag{SI.21}$$

For each exponential in the intermediate scattering function, we obtain the following Lorentzian from the Fourier transform, i.e.,

$$\mathcal{F}\left[\exp\left(-t\Gamma\right)\right] = \frac{1}{\pi}\frac{\Gamma}{\omega^2+\Gamma^2}, \tag{SI.22}$$

and for $l=0$ we get the elastic line, $j_0^2(QR)\delta(\omega)$, where $\delta(\omega)$ is a Dirac delta-function. Hence, the total dynamic structure factor is,

$$\begin{aligned} S(Q,\omega) = \frac{1}{2\pi}\Bigg(& j_0^2(QR)\delta(\omega) + 3j_1^2(QR)\left[\frac{1}{\pi}\frac{D_s+D_t}{\omega^2+(D_s+D_t)^2}\right] \\ & + 5j_2^2(QR)\left[\frac{1}{4}\frac{1}{\pi}\frac{6D_t}{\omega^2+(6D_t)^2} + \frac{3}{4}\frac{1}{\pi}\frac{4D_s+2D_t}{\omega^2+(4D_s+2D_t)^2}\right]\Bigg). \end{aligned} \tag{SI.23}$$

We rewrite this in the form of different Γ_x values in Eq. 3.

SI.VI: Rotational Autocorrelation Function Analysis

The first 1 ns of the production simulation trajectory was taken, and the rotational autocorrelation functions perpendicular and parallel to the principal axis of rotation were found for values of $t = (0 \text{ to } 7.5)$ ps. These were expressed with the second-order Legendre polynomial, P_2, of the angle $\alpha(t)$, between a molecular axis at time t and its initial orientation at $t=0$,

$$G_\theta(t) = \langle P_2\left[\cos\left\{\alpha(t)\right\}\right]\rangle = \left\langle \frac{1}{2}\left[3\cos^2\left\{\alpha(t)\right\}-1\right]\right\rangle, \tag{SI.24}$$

where θ defines the angle between the vector and the principal axis. The resulting rotational autocorrelation functions can be found in Fig. 2.

To extract the tumbling and spinning rotational diffusion coefficients, the data were modelled simultaneously using Eq. SI.5 and Eq. SI.6, augmented by additional Gaussian or exponential decay terms to account for dynamics not described by these models. Bayesian model selection, via nested sampling [21] using the DYNESTY [22], was used to determine how many additional Gaussian and exponential decay functions were necessary to fully describe the observed data, with the Bayesian evidence values given in Table SI.1.

TABLE SI.1. Log-Bayesian evidence values for different models used to describe the observed rotational autocorrelation function, the model with the highest Bayesian evidence shown in bold (the number of parameters for each model is given in square brackets). In the column where 0 is added to $G_{90^\circ}(t)$, $A_\mathcal{N}$ is a normalisation applied to $G_{0^\circ}(t)$ or $G_{90^\circ}(t)$, and $\mathcal{N}(0,\sigma)$ indicates a Gaussian distribution, centred on 0 with a standard deviation of σ.

$G_{0^\circ}(t)$ \ $G_{90^\circ}(t)$	$+0$	$+(1-A_\mathcal{N})\mathcal{N}(0,\sigma)$
$+(1-A_1)\exp\left(-{}^{t}/_{\tau_1}\right)$	1103.3 [4]	1129.7 [6]
$+A_2\exp\left(-{}^{t}/_{\tau_1}\right)+(1-A_1-A_2)\mathcal{N}(0,\sigma)$	1120.6 [6]	1140.6 [8]
$+A_2\exp\left(-{}^{t}/_{\tau_1}\right)+(1-A_1-A_2)\exp\left(-{}^{t}/_{\tau_2}\right)$	1124.4 [6]	**1144.3** [8]
$+A_2\exp\left(-{}^{t}/_{\tau_1}\right)+A_3\exp\left(-{}^{t}/_{\tau_2}\right)+(1-A_1-A_2-A_3)\mathcal{N}(0,\sigma)$	1122.6 [8]	1142.4 [10]

The highest-evidence model consisted of Eq. SI.6 with an additional Gaussian distribution to account for very fast dynamics,

$$G_{90^\circ}(t) = A_n\left[\frac{3}{4}\exp\left(-t(4D_s+2D_t)\right)+\frac{1}{4}\exp\left(-t6D_t\right)\right]+(1-A_\mathcal{N})\mathcal{N}(0,\sigma), \tag{SI.25}$$

and Eq. SI.5 with two additional exponential decays,

$$G_{0^\circ}(t) = A_1 \exp\left(-t6D_t\right) + A_2 \exp\left(\frac{-t}{\tau_1}\right) + \left(1 - A_1 - A_2\right) \exp\left(\frac{-t}{\tau_2}\right), \tag{SI.26}$$

The full posterior distribution for this model is shown, as a corner plot, in Fig. SI.2. The fast decay, with time constant $\tau_1 = 0.21\,\mathrm{ps}$, is related to the dramatic decrease observed in the Bayesian evidence at $1.5\,\mathrm{meV}$ (corresponding to around $(0.43$ to $0.52)\,\mathrm{ps}$). This further justifies the argument made in the main text that it is the presence of fast motions, not present in either analytical model, which leads to the decrease in Bayesian evidence.

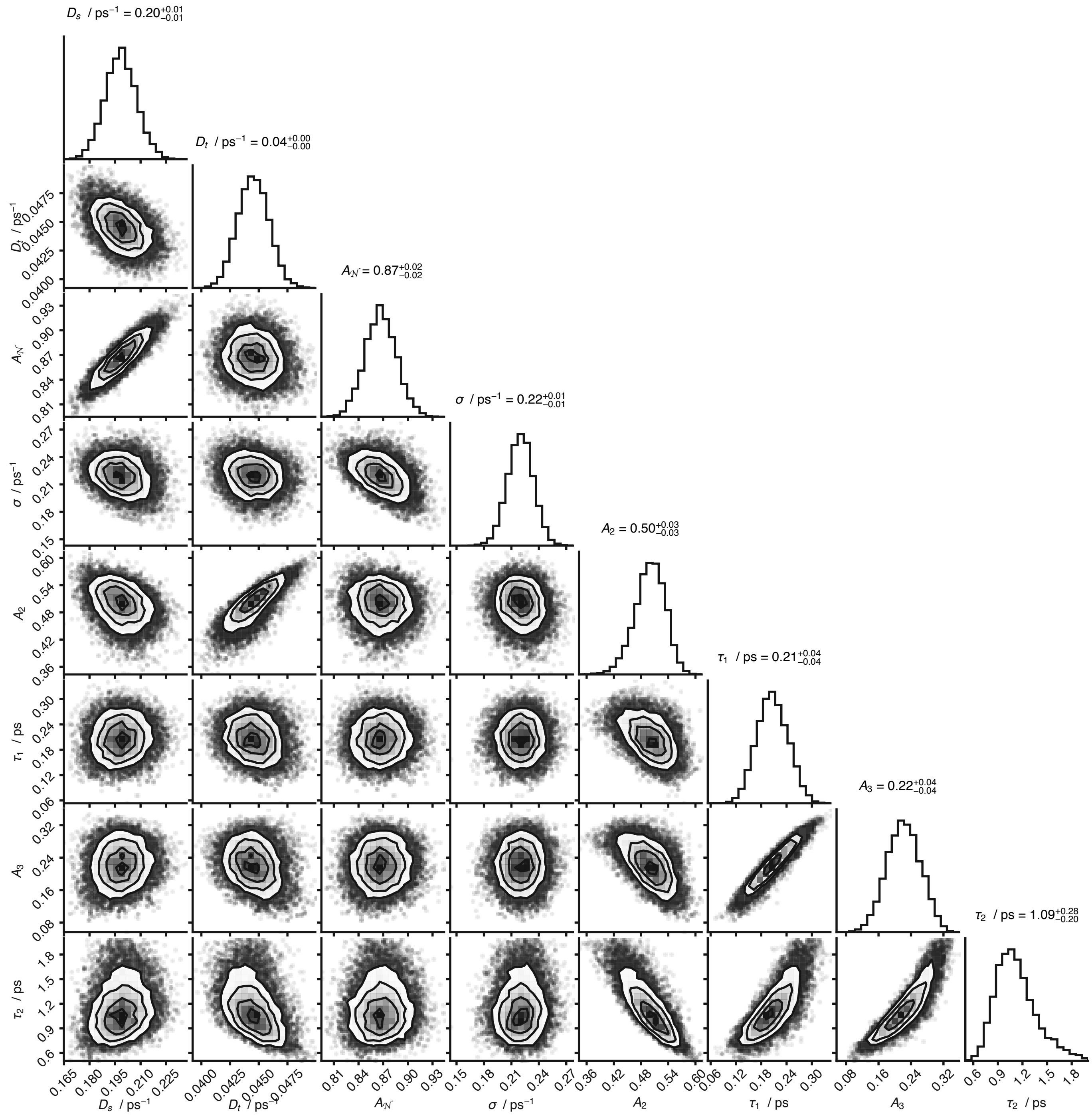

FIG. SI.2. Corner plot showing the posterior distribution of parameters for the highest evidence model given the rotational autocorrelation function data.

SI.VII: Comparison with Analysis Used in Previous Literature

Previous works found a pair of effective characteristic times, $\tau_{\mathrm{eff},\theta}$, by fitting stretched exponential functions to the rotational autocorrelation functions for $\theta = 90°$ and $\theta = 0°$ and computing the integrals with respect to correlation time, t [20, 23–26]. These integrals can then be used to find D_s and D_t by solving the system of equations,

$$\tau_{\mathrm{eff},90^\circ} = \int_0^\infty G_{90^\circ}(t)\,\mathrm{d}t = \frac{3}{4}\frac{1}{4D_s + 2D_t} + \frac{1}{4}\frac{1}{6D_t}, \tag{SI.27}$$

and

$$\tau_{\mathrm{eff},0^\circ} = \int_0^\infty G_{0^\circ}(t)\,\mathrm{d}t = \frac{1}{6D_t}. \tag{SI.28}$$

In practice, this is achieved by fitting a stretched exponential of the form,

$$G_\theta(t) = \exp\left[-\left(\frac{t}{\alpha}\right)^\beta\right], \tag{SI.29}$$

where α and β are fitting parameters, the integral is then found as,

$$\tau_{\mathrm{eff},\theta} = \frac{\alpha_\theta}{\beta_\theta}\Gamma\left(\frac{1}{\beta_\theta}\right). \tag{SI.30}$$

This approach introduces a subtle inconsistency: Eq. SI.6 describes, for $\theta = 0°$, the correlation function as the sum of two exponential functions with different decay rates, whereas the application of the integral at the timescale yields a single effective timescale. This inconsistency brings doubt into the previously determined values of D_s and D_t.

Applying the approach taken in the literature to our simulation data, gives effective characteristic times of $\tau_{\mathrm{eff},90^\circ} = 1.49\,\mathrm{ps}$ and $\tau_{\mathrm{eff},0^\circ} = 2.33\,\mathrm{ps}$. Using Eqs. SI.27 and SI.28, we can find rotational diffusion coefficients of $D_t = 0.07\,\mathrm{ps}^{-1}$ and $D_s = 0.17\,\mathrm{ps}^{-1}$. These are in good agreement with the literature values presented in previous work [20, 24–26]. However, to our simulation data, we can apply Bayesian model selection to determine whether there is sufficient evidence for the more complex Eq. 1 over the conceptually simpler stretched-exponential function. The log-Bayesian evidence for our analytical model for the rotational autocorrelation functions is 1144.3, compared to 1052.5 for fitting a stretched exponential function to each rotational autocorrelation function, i.e., there is strong support for the more complex Eq. 1.

SI.VIII: QENS Signal from Decomposed Trajectory

To generate the rotational-only simulation trajectory, each benzene molecule was re-centred relative to its molecular centre of mass at $t = 0\,\mathrm{ns}$ for every frame within the full trajectory, with the centre of mass computed via the *pseudo* centre-of-mass recentering method [14]. This molecular recentring was applied at each simulation time step in the trajectory, removing all translational motion while preserving molecular rotation. A complementary translational simulation trajectory was constructed by rigidly fixing the intra-molecular coordinates of each benzene molecule relative to its molecular centre of mass at $t = 0\,\mathrm{ns}$, resulting in a trajectory containing only the translational motion of each molecule.

Total and incoherent-only dynamic structure factors for both trajectories were computed using MDANSE [27]. Example spectra for the rotational and translational components are shown in Fig. SI.3a. The rotational spectrum exhibits an elastic contribution, represented by a Dirac δ-function, arising from the static spatial correlations in benzene's molecular structure. The amplitude of this elastic component follows the decay of the zeroth-order Bessel function evaluated at the molecular radius of benzene ($\sim 2.48\,\text{Å}$) as a function of Q, in agreement with theoretical expectations [28], as illustrated in Fig. SI.3b. The independent spectra can be re-convolved to reconstruct the incoherent simulated spectra within the dynamical range of interest with minimal loss, as shown in Figs. SI.3c and SI.3d. The only notable discrepancy occurs at very high Q, where the re-convolved spectrum reproduces the correct shape but underestimates the amplitude. This discrepancy may be attributed to numerical effects, as the signal intensity in this region is extremely low, or unaccounted for roto-translational coupling.

Models were fit to the rotation-only spectra produced from the decomposed rotational trajectory. To determine whether a flat background term was required at each Q, the Bayesian evidence was found for a series of 13 models given data, which was binned into the same 12 Q-bins as the IRIS data, progressively increasing the number of flat

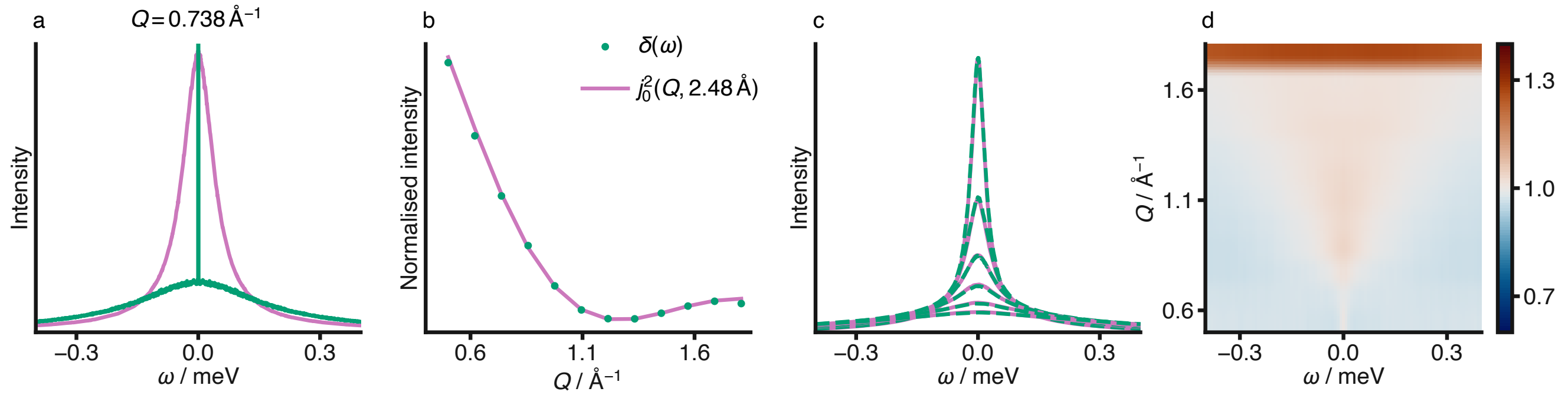

FIG. SI.3. Simulation decomposition: (a) Exemplary rescaled translational (pink) and rotational (green) spectra demonstrating the structural delta peak in the rotational spectra at $Q = 0.738\,\text{Å}^{-1}$, (b) agreement between the decay of the structural δ-function with the zeroth order Bessel function ($R = 2.48\,\text{Å}$) (c) Comparison of the first 6 simulated incoherent spectra (pink) ($Q_{\text{mid}} = (0.50,\ 0.62,\ 0.74,\ 0.86,\ 0.98,\ \text{and}\ 1.10)\,\text{Å}^{-1}$) and the re-convolved decomposed spectra (green dashed). (d) Ratio of full simulated incoherent spectra to re-convolved decomposed spectra.

background components. Background terms were introduced sequentially from the highest to the lowest Q. This ordering reflects the fact that larger Q-values probe shorter length scales and therefore faster dynamics, which are more likely to lie outside the instrumental ω-dynamic range of the IRIS spectrometer ($\pm 0.4\,\text{meV}$) and manifest as an approximately flat contribution to the spectra. For both the isotropic and the anisotropic model, there was the sufficient evidence to fit 6 background terms at ($Q_{\text{mid}} = (1.21,\ 1.33,\ 1.45,\ 1.57,\ 1.69,\ \text{and}\ 1.81)\,\text{Å}$) with log-Bayesian evidences of -99.5 and -71.0 respectively, justifying the additional complexity of the anisotropic model.

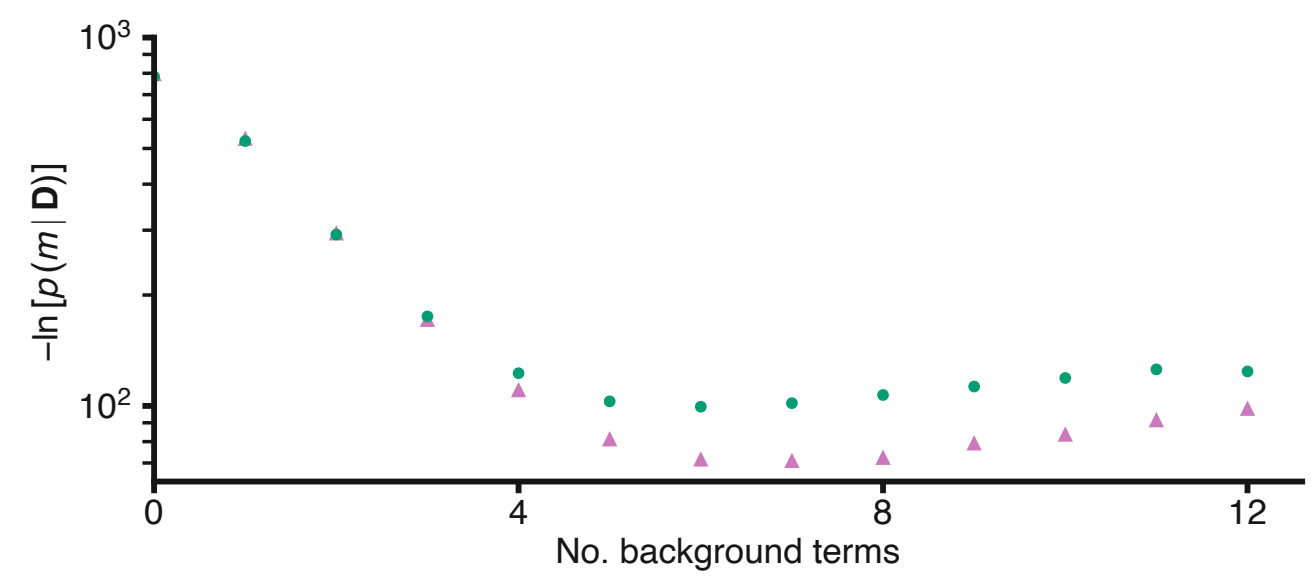

FIG. SI.4. The negative log-Bayesian evidence for the isotropic (green dots) and anisotropic (pink triangles) models for the decomposed rotational QENS spectra as a function of the number of background terms included across Q at $\pm 0.5\,\text{meV}$ dynamic range.

SI.IX: Model fitting

The full model that was assessed, given the experimental data, was

$$S_{\text{inc}}(Q,\omega) = C_{\text{norm}} \left[\left(e^{-Q^2 \langle u^2 \rangle /3} \left[\frac{1}{\pi} \frac{D^* Q^2}{\omega^2 + (D^* Q^2)^2} \right] \otimes S_{\text{R}}(Q,\omega) + bkg \right) \otimes S_{\text{res}}(Q,\omega) \right], \tag{SI.31}$$

where, bkg indicates a flat background describing fast motions beyond the resolution of the instrument, C_{norm} is a normalisation term, $S_{\text{R}}(Q,\omega)$ is either the anisotropic model, Eq. 3, or the isotropic model,

$$S_R(Q,\omega) = j_0^2(QR)\,\delta(\omega) + 3\,j_1^2(QR) \left[\frac{1}{\pi} \frac{2D_r}{\omega^2 + (2D_r)^2} \right] + 5\,j_2^2(QR) \left[\frac{1}{\pi} \frac{6D_r}{\omega^2 + (6D_r)^2} \right], \tag{SI.32}$$

and S_{res} is the appropriate resolution function data, normalised by the sum of its intensity.

Nested sampling was performed for the model, given the data at incident energies (3.60 and 1.97) meV, simultaneously. As such, when the nested sampling was performed, the model in Eq. SI.31 had either 3 or 4 global parameters for the isotropic or anisotropic models, respectively (D^*, $\langle u^2 \rangle$, D_s, D_t, and $D_r = D_t = D_s$ for the isotropic). A single $\langle u^2 \rangle$ was used for both incident energies; this parameter describes motions faster than ω-dynamic range and appears

as a flat background [29]. E_i-dependent parameters, including a flat background, were included at each Q-value for each incident energy in both models. A constant normalisation term, C_{norm}, was fitted for each incident energy. C_{norm} was used to normalise across Q for each incident energy, to account for the non-conservation of spectral area introduced by the fast Fourier transform, and to scale to the experimental intensities. To fulfil the characteristics of a Dirac delta function (integrates to 1), the delta function intensity at $Q = 0$ was defined as $\delta(\omega)^{-1}$. For all models, the radius of gyration R was fixed to the radius of the benzene molecule (2.48 Å). All model priors were uniform priors of the form $\theta \in [\theta_{\min}, \theta_{\max})$, where $\theta_{\min}$ and $\theta_{\max}$ for each parameter are given in Table SI.2.

TABLE SI.2. The model prior minimum and maximum values for each parameter.

	D^*	D_r	D_t	D_s	$\langle u \rangle$	C_{norm}	bkg
θ_{min}	0.01	0.01	0.001	0.06	0.001	0.01	0.01
θ_{max}	0.2	1	0.15	1	0.18	30	1.5

Due to the distributivity of addition and convolution, the convolution of the rotational and translational Lorentzian components and the Dirac δ-function can be written analytically as a sum of Lorentzian functions. In the limit of infinite energy range and continuous resolution, this analytical expression is mathematically identical to a numerical fast Fourier transform-based convolution. However, in reality, where no analytical resolution function is available, the experimental spectra were measured over a finite energy window and sampled on a discrete energy grid. The Lorentzian tails are therefore truncated, and the convolution becomes discrete rather than continuous, breaking the exact equivalence between the analytic and fast Fourier transform convolutions. Because the fast Fourier transform convolution naturally incorporates these finite-window and discretisation effects, it provided a representation that more closely matched the measured data. For this reason, the fast Fourier transform convolution approach, using `scipy.signal.fftconvolve`, was used for model fitting.

SI.X: QENS Model Agreement

The anisotropic model for the incoherent dynamic structure factor was modelled simultaneously, given the incoherent QENS signal at incident energies of (3.60 and 1.97) meV, with a ω-dynamic range ± 1.25 meV. This modelling yielded the marginal posterior distributions shown in Fig. 6. The correlation corner plot for these marginal posterior distributions is shown in Fig. SI.5; as expected, D_t and D_s are slightly correlated. The marginal posterior distributions of the other key parameters, $\langle u^2 \rangle$ and the flat background, as a function of Q for each incident energy are shown in Fig. SI.6.

The model agreement, represented by the maximum *a posteriori* for each of the incident energies, is shown in Fig. SI.7 (3.6 meV, 12 Q bins) and Fig. SI.8 (1.97 meV, 14 Q bins), indicating very good agreement between the model and the data across all Q for both incident energies. In the interest of clarity, we compare the maximum *a-posteriori* for the isotropic and anisotropic models with the data in Figs. SI.7 & SI.8. There appears to be some structuring present in the ratio between the maximum *a-posteriori* isotropic model and the data (specifically the model underestimates the dynamic structure factor at small ω and $1.1\,\text{Å}^{-1} \le Q < Q_{\max}$), which is not observed for the anisotropic model.

SI.XI: Position and Orientation Probability Density Function

The relative orientations of pairs of benzene molecules were evaluated as a function of their centre-to-centre separation for 2000 randomly chosen frames from the full benzene simulation trajectory (Fig. SI.9). The intermolecular angle was defined as the angle between vectors parallel to the principal axis of rotation of the benzene molecule, as defined by Falkowska *et al.* [30]. The angles were found for molecules between (3 to 8) Å (in 34 bins) and binned into 45 evenly spaced angular bins between (0 to 90)°. The resulting two–dimensional histogram $g(r, \theta)$ was normalised by the bulk number density and the spherical sector volume corresponding to each (r, θ) bin,

$$V_{bin} = \frac{2\pi}{3}\left(r_{i+1}^3 - r_i^3\right)\left(\cos\theta_j - \cos\theta_{j+1}\right), \tag{SI.33}$$

The radial distribution function $g(r)$ and angular distribution function $g(\theta)$ were obtained by numerically integrating $g(r, \theta)$ over θ and r, respectively.

From the experimental analysis of total neutron scattering data from benzene, Falkowska *et al.* observed the presence of tightly bound (< 4 Å) π-stacked benzene dimers and perpendicularly structured dimers in either 'T' or

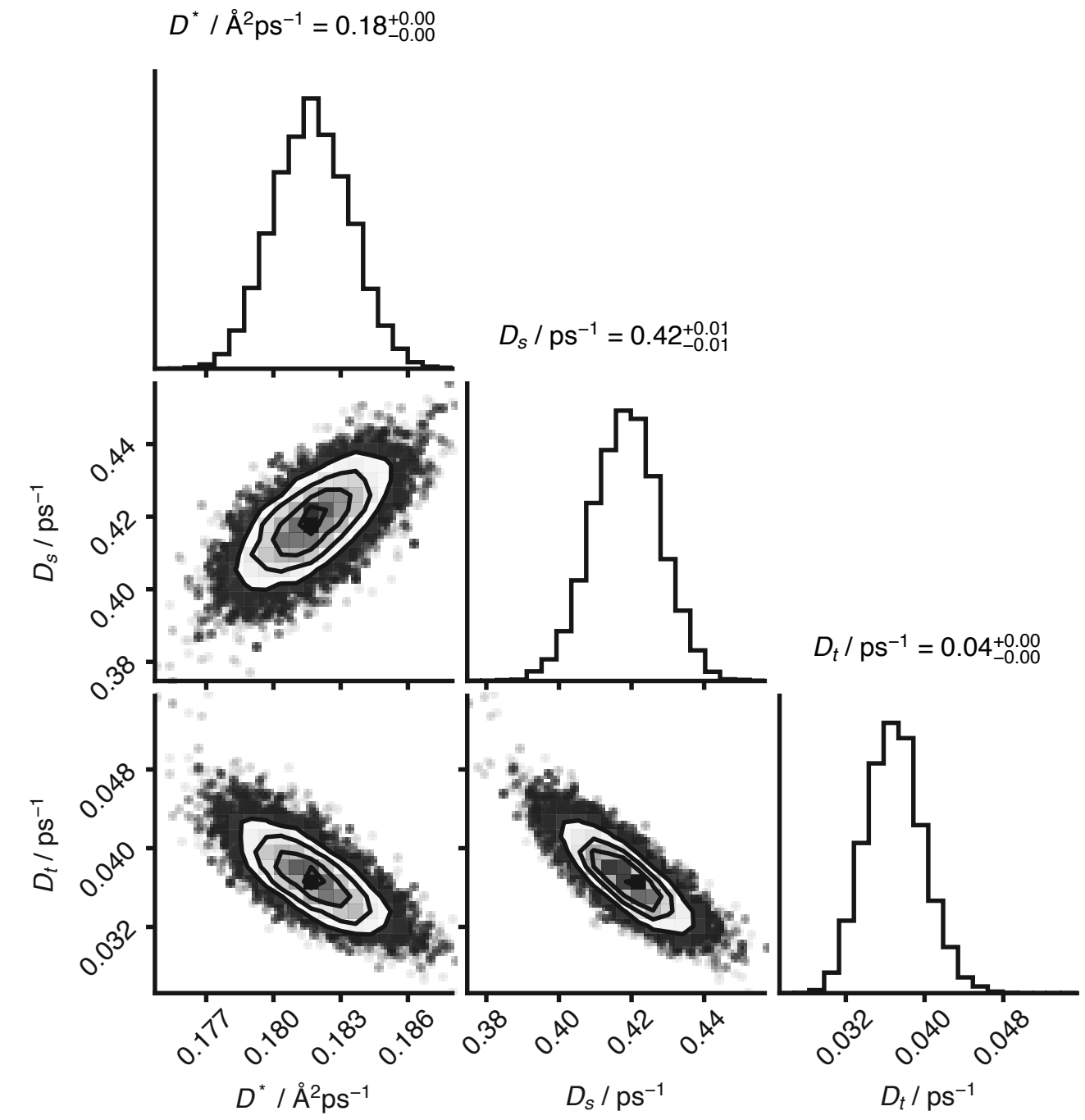

FIG. SI.5. Correlation plot of the Fickian diffusion coefficient, D^* and the spinning, D_s and tumbling, D_t, rotational diffusion coefficients from the anisotropic model given the experimental data.

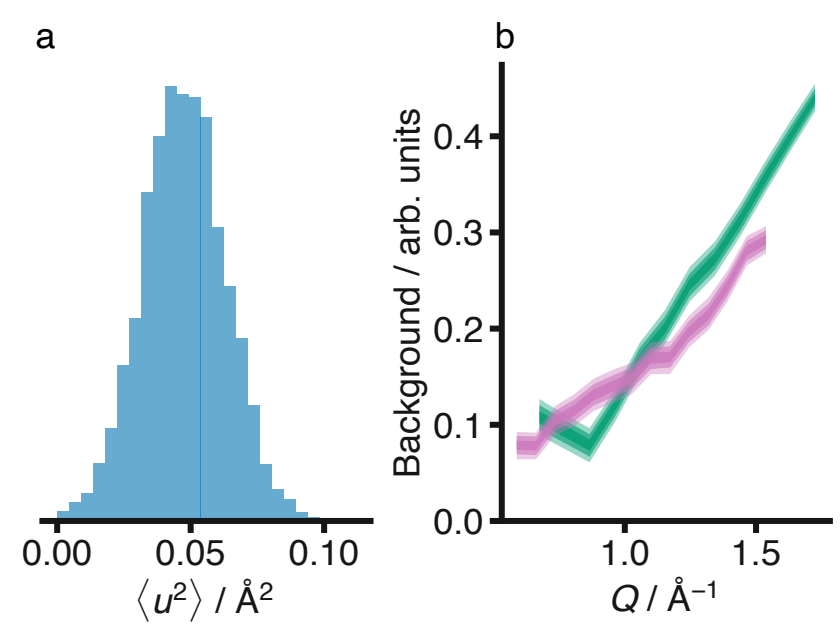

FIG. SI.6. Marginal posterior distributions for (a) the mean-squared displacement (MSD) parameter $\langle u^2 \rangle$, in the Debye–Waller factor in the anisotropic model and (b) the flat background intensity as a function of Q at incident energies of 3.60 meV (green) and 1.97 meV (pink), shading indicates the 1σ, 2σ, and 3σ credible intervals.

'Y' configurations ($70° < \theta \leq 90°$, see Fig. 14 in ref. [30]). In our simulations, we observe some presence of the 'T' and 'Y' configurations, but much less than in the experiment, i.e., no intensity increase at high θ. Therefore, we do not see evidence of the tightly bound π-stacked structures. We believe that the presence of such dimeric structures may be related to the greater rotational anisotropy observed in our experiment than in the simulation analysis.

REFERENCES

[1] Thompson, A. P.; Aktulga, H. M.; Berger, R.; Bolintineanu, D. S.; Brown, W. M.; Crozier, P. S.; in 't Veld, P. J.; Kohlmeyer, A.; Moore, S. G.; Nguyen, T. D.; Shan, R.; Stevens, M. J.; Tranchida, J.; Trott, C.; Plimpton, S. J. LAMMPS - a flexible simulation tool for particle-based materials modeling at the atomic, meso, and continuum scales. *Comput. Phys. Comm.* **2022**, *271*, 108171, doi:10.1016/j.cpc.2021.108171.

[2] Dodda, L. S.; Cabeza de Vaca, I.; Tirado-Rives, J.; Jorgensen, W. L. LigParGen web server: an automatic OPLS-AA parameter generator for organic ligands. *Nucleic Acids Res.* **2017**, *45*, W331–W336, doi:10.1093/nar/gkx312.

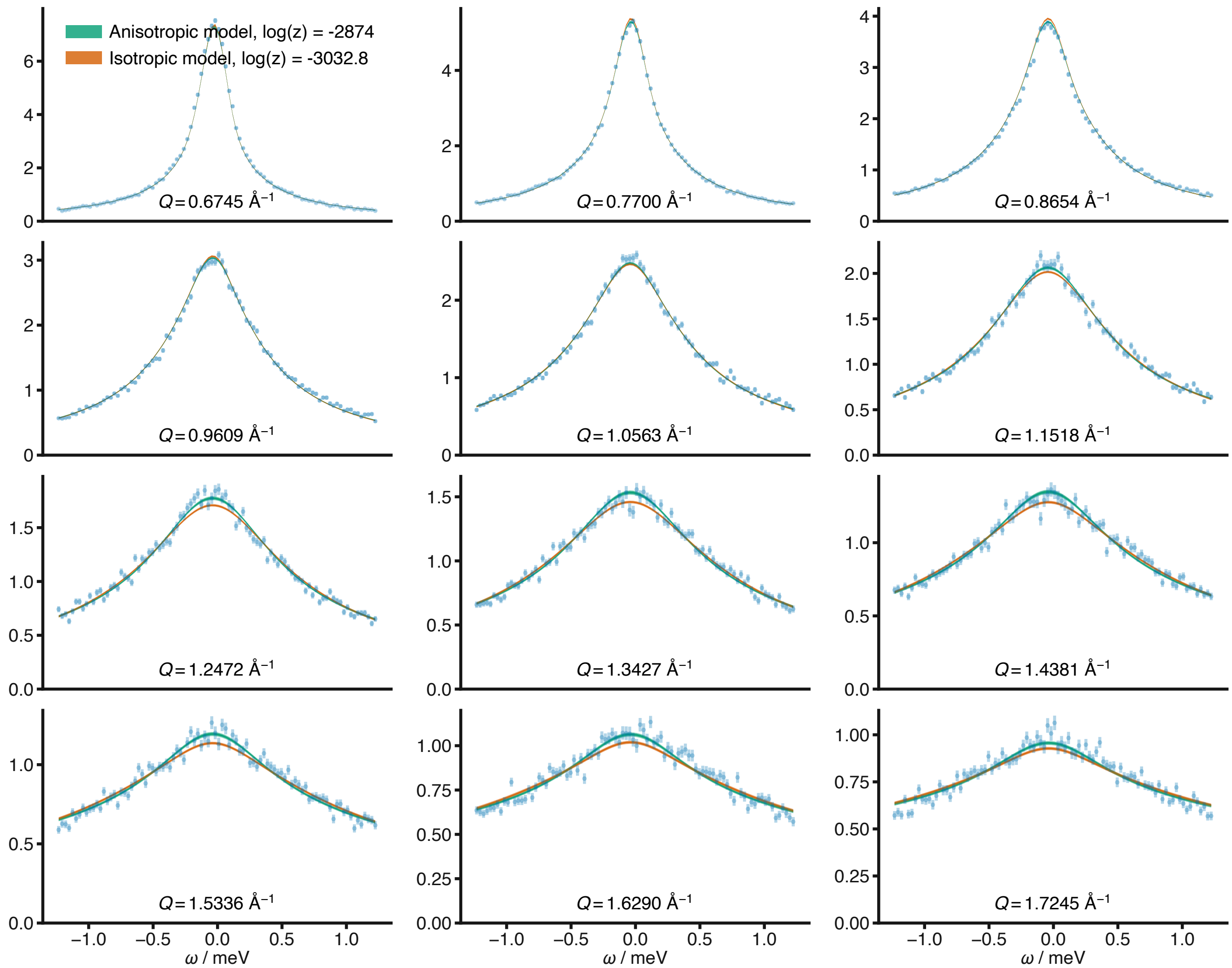

FIG. SI.7. Comparison between the maximum *a posteriori* for the anisotropic model (green) given the binned p-LET data (blue, with errorbars indicating a single standard deviation) at an incident energy of 3.60 meV, at the 12 Q-bins used.

[3] Jorgensen, W. L.; Maxwell, D. S.; Tirado-Rives, J. Development and Testing of the OPLS All-Atom Force Field on Conformational Energetics and Properties of Organic Liquids. *J. Am. Chem. Soc.* **1996**, *118*, 11225–11236, doi:10.1021/ja9621760.

[4] Eastwood, J.; Hockney, R.; Lawrence, D. P3M3DP-the three-dimensional periodic particle-particle/particle-mesh program. *Comput. Phys. Commun.* **1984**, *35*, C–618–C–619, doi:10.1016/S0010-4655(84)82783-6.

[5] Nosé, S. A unified formulation of the constant temperature molecular dynamics methods. *J. Chem. Phys.* **1984**, *81*, 511–519, doi:10.1063/1.447334.

[6] Hoover, W. G. Canonical dynamics: Equilibrium phase-space distributions. *Phys. Rev. A* **1985**, *31*, 1695–1697, doi:10.1103/physreva.31.1695.

[7] Martyna, G. J.; Klein, M. L.; Tuckerman, M. Nosé–Hoover chains: The canonical ensemble via continuous dynamics. *J. Chem. Phys.* **1992**, *97*, 2635–2643, doi:10.1063/1.463940.

[8] Martyna, G. J.; Tobias, D. J.; Klein, M. L. Constant pressure molecular dynamics algorithms. *J. Chem. Phys.* **1994**, *101*, 4177–4189, doi:10.1063/1.467468.

[9] Malek, N. I.; Ijardar, S. P.; Master, Z. R.; Oswal, S. B. Temperature dependence of densities, speeds of sound, and derived properties of cyclohexylamine+cyclohexane or benzene in the temperature range 293.15–323.15K. *Thermochimica Acta* **2012**, *547*, 106–119, doi:10.1016/j.tca.2012.08.011.

[10] Arbe, A.; Nilsen, G. J.; Stewart, J. R.; Alvarez, F.; Sakai, V. G.; Colmenero, J. Coherent structural relaxation of water from meso- to intermolecular scales measured using neutron spectroscopy with polarization analysis. *Phys. Rev. Res.* **2020**, *2*, 022015, doi:10.1103/PhysRevResearch.2.022015.

[11] Cassella, G.; Stewart, J. R.;釘Paternò, G. M.; García Sakai, V.; Devonport, M.; Galsworthy, P. J.; Bewley, R. I.; Voneshen, D. J.; Raspino, D.; Nilsen, G. J. Polarization analysis on the LET cold neutron spectrometer using a ^{3}He

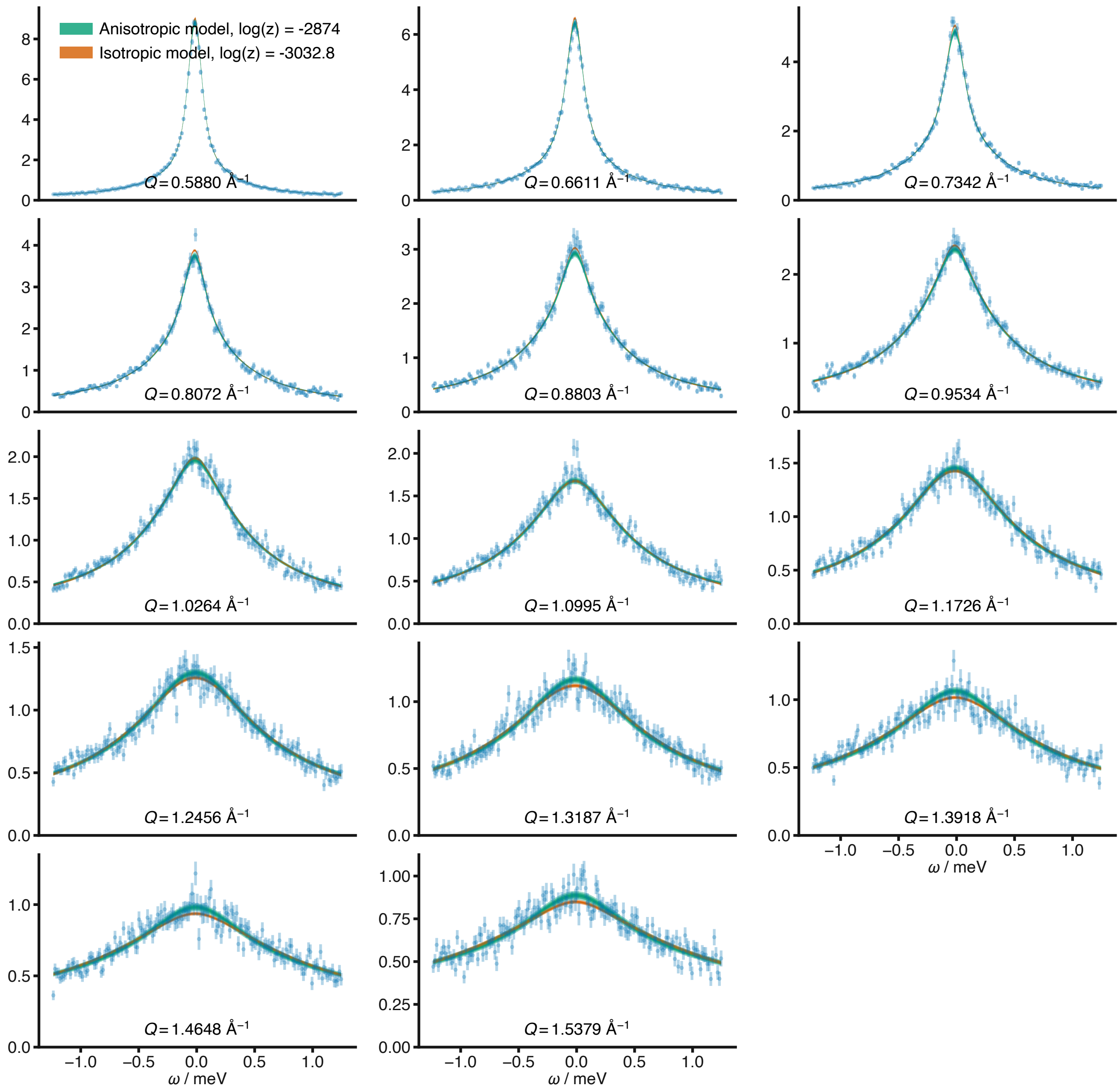

FIG. SI.8. Comparison between the maximum *a posteriori* for the anisotropic model (green) and isotropic model (orange) given the binned p-LET data (blue, with errorbars indicating a single standard deviation) at an incident energy of 1.97 meV, at the 14 Q-bins used.

spin-filter: first results. *J. Phys.: Conf. Ser.* **2019**, *1316*, 012007, doi:10.1088/1742-6596/1316/1/012007.

[12] De Gennes, P. Liquid dynamics and inelastic scattering of neutrons. *Physica* **1959**, *25*, 825–839, doi:10.1016/0031-8914(59)90006-0.

[13] McCluskey, A. R.; Squires, A. G.; Dunn, J.; Coles, S. W.; Morgan, B. J. kinisi: Bayesian analysis of mass transport from molecular dynamics simulations. *J. Open Source Softw.* **2024**, *9*, 5984, doi:10.21105/joss.05984.

[14] Richardson, H.; Dunn, J.; McCluskey, A. R. On the estimation of center of mass in periodic systems. *J. Chem. Phys.* **2025**, *162*, 204103, doi:10.1063/5.0260928.

[15] McCluskey, A. R.; Coles, S. W.; Morgan, B. J. Accurate Estimation of Diffusion Coefficients and their Uncertainties from Computer Simulation. *J. Chem. Theory Comput.* **2024**, *21*, 79–87, doi:10.1021/acs.jctc.4c01249.

[16] Favro, L. D. Theory of the Rotational Brownian Motion of a Free Rigid Body. *Phys. Rev.* **1960**, *119*, 53–62,

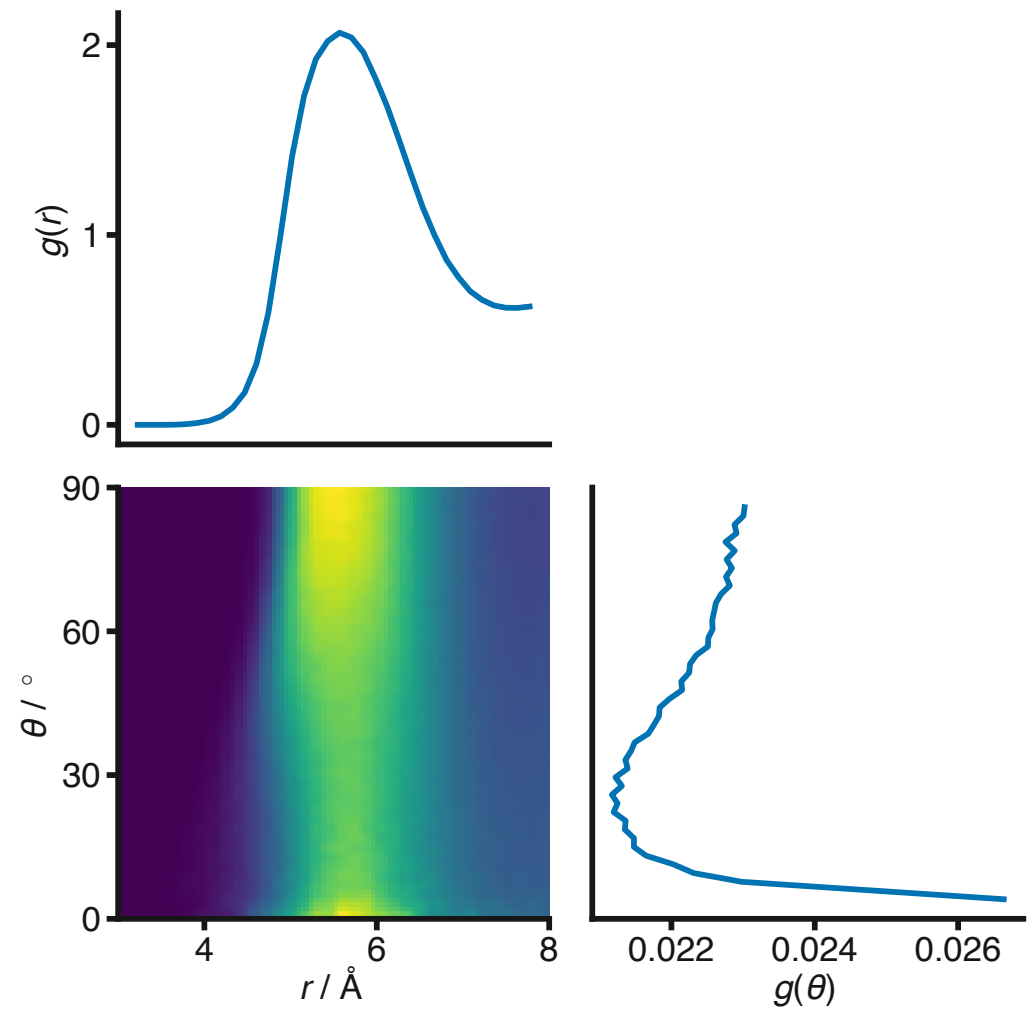

FIG. SI.9. Orientational selectivity by distance showing the centre-to-centre radial distribution function, angular distribution function of molecular pairs and the correlation plot between them.

doi:10.1103/physrev.119.53.

[17] These are analogous to the l and m_l orbital angular and magnetic quantum numbers.

[18] Wigner, E. P. *Group Theory and Its Applications to the Quantum Mechanics of Atomic Spectra*, 4th ed.; Academic Press: New York, US, 1964.

[19] Woessner, D. E. Molecular Reorientation in Liquids. Deuteron Quadrupole Relaxation in Liquid Deuterium Oxide and Perdeuterobenzene. *J. Chem. Phys.* **1964**, *40*, 2341–2348, doi:10.1063/1.1725512.

[20] Witt, R.; Sturz, L.; Dölle, A.; Müller-Plathe, F. Molecular Dynamics of Benzene in Neat Liquid and a Solution Containing Polystyrene. ^{13}C Nuclear Magnetic Relaxation and Molecular Dynamics Simulation Results. *J. Phys. Chem. A* **2000**, *104*, 5716–5725, doi:10.1021/jp000201p.

[21] Skilling, J. Nested Sampling. AIP Conference Proceedings. 2004; p 395–405, doi:10.1063/1.1835238.

[22] Speagle, J. S. dynesty: a dynamic nested sampling package for estimating Bayesian posteriors and evidences. *Mon. Not. R. Astron. Soc* **2020**, *493*, 3132–3158, doi:10.1093/mnras/staa278.

[23] Gillen, K. T.; Griffiths, J. E. Molecular reorientations in liquid benzene: raman line shapes and 2D NMR relaxation. *Chem. Phys. Lett.* **1972**, *17*, 359–364, doi:10.1016/0009-2614(72)87096-9.

[24] Laaksonen, A.; Stilbs, P.; Wasylishen, R. E. Molecular motion and solvation of benzene in water, carbon tetrachloride, carbon disulfide and benzene: A combined molecular dynamics simulation and nuclear magnetic resonance study. *J. Chem. Phys.* **1998**, *108*, 455–468, doi:10.1063/1.475408.

[25] Schwartz, M.; Duan, D.; Berry, R. J. Molecular Dynamics Study of Anisotropic Translational and Rotational Diffusion in Liquid Benzene. *J. Phys. Chem. A* **2005**, *109*, 8637–8641, doi:10.1021/jp0530799.

[26] Fu, C.-F.; Tian, S. X. A Comparative Study for Molecular Dynamics Simulations of Liquid Benzene. *J. Chem. Theory Comput.* **2011**, *7*, 2240–2252, doi:10.1021/ct2002122.

[27] Goret, G.; Aoun, B.; Pellegrini, E. MDANSE: An Interactive Analysis Environment for Molecular Dynamics Simulations. *J. Chem. Inf. Model.* **2017**, *57*, 1–5, doi:10.1021/acs.jcim.6b00571.

[28] Johnson, M. J.; Kearley, G. J. In *Neutron Scattering – Fundamentals*; Fernandez-Alonso, F., Price, D. L., Eds.; Experimental Methods in the Physical Sciences; Elsevier, 2013; Vol. 44; p 415–469, doi:10.1016/b978-0-12-398374-9.00007-3.

[29] Bée, M. *Quasielastic neutron scattering, principles and applications in solid state chemistry, biology and materials science*; CRC Press: London, UK, 1988.

[30] Falkowska, M.; Bowron, D. T.; Manyar, H. G.; Hardacre, C.; Youngs, T. G. A. Neutron Scattering of Aromatic and Aliphatic Liquids. *ChemPhysChem* **2016**, *17*, 2043–2055, doi:10.1002/cphc.201600149.
